# 2D‐Nanofiller‐Based Polymer Nanocomposites for Capacitive Energy Storage Applications

**DOI:** 10.1002/smsc.202300016

**Published:** 2023-04-25

**Authors:** Sumit Bera, Maninderjeet Singh, Rukshan Thantirige, Saurabh Kr Tiwary, Brian T. Shook, Elianie Nieves, Dharmaraj Raghavan, Alamgir Karim, Nihar R. Pradhan

**Affiliations:** ^1^ Department of Chemistry, Physics and Atmospheric Science Jackson State University 1400 John R. Lynch Street Jackson MS 392017 USA; ^2^ Department of Chemical and Biomolecular Engineering University of Houston TX 77204 USA; ^3^ Department of Chemistry Howard University Washington DC WA 20059 USA

**Keywords:** 2D materials, composites, high-energy-density, polymers, thin films

## Abstract

High‐energy‐density storage devices play a major role in modern electronics from traditional lithium‐ion batteries to supercapacitors for a variety of applications from rechargeable devices to advanced military equipment. Despite the mass adoption of polymer capacitors, their application is limited by their low energy densities and low‐temperature tolerance. Polymer nanocomposites based on 2D nanomaterials have superior capacitive energy densities, higher thermal stabilities, and higher mechanical strength as compared to the pristine polymers and nanocomposites based on 0D or 1D nanomaterials, thus making them ideal for high‐energy‐density dielectric energy storage applications. Here, the recent advances in 2D‐nanomaterial‐based nanocomposites and their implications for energy storage applications are reviewed. Nanocomposites based on conducting 2D nanofillers such as graphene, reduced graphene oxide, MXenes, semiconducting 2D nanofillers including transition metal dichalcogenides such as MoS_2_, dielectric 2D nanofillers including hBN, Mica, Al_2_O_3_, TiO_2_, Ca_2_Nb_3_O_10_ and MMT, and their effects on permittivity, dielectric strength, capacitive energy density, efficiency, thermal stability, and the mechanical strength, are discussed. Also, the theory and machine‐learning‐guided design of polymer 2D nanomaterial composites is learnt and the challenges and opportunities for developing ultrahigh‐capacitive‐energy‐density devices based on these nanofiller polymer composites are presented.

## Graphical abstract

2D‐nanomaterials‐based polymer nanocomposites have revolutionized the field of capacitive energy storage by enhancing the dielectric, thermal, and mechanical properties of pristine polymers. The effects of 2D nanofillers on dielectric and capacitive energy storage properties of polymer nanocomposites are summarized in detail in context to relevant applications and challenges and opportunities for future research in these materials are described.

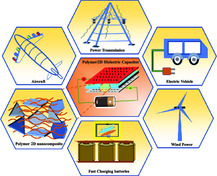

## 1 Introduction

The development of high‐energy‐density energy storage devices has received great attention in recent years as the demand for renewable and sustainable energy sources continues to increase. Traditional energy storage devices, such as batteries, have limited energy density and are not suitable for certain applications where fast charging and discharging are required. Despite being able to produce a high current through fast charging and discharging, capacitors with pristine polymers, cyclic, and copolymers inherit low energy densities,^[^ ^1^, ^2^, ^3^ ^]^ making them less desirable in energy storage applications. To address this issue, researchers have been exploring the use of polymer and 2D nanofiller composites as a means of increasing energy density^[^ ^4^, ^5^ ^]^ and permittivity.^[^ ^6^ ^]^ Polymer materials have long been used in energy storage devices due to their low cost, lightweight, and flexibility. However, their energy density is typically low, making them less suitable for certain applications. By incorporating 2D nanofillers, such as graphene or MoS_2_, into the polymer matrix, it is possible to significantly increase the energy density of the composite material. In this case, 2D nanofillers act as conductive pathways, allowing for faster charge and discharge rates. Yang et al.^[^ ^7^ ^]^ reported improved energy densities up to 17.33 J cm^−3^ in composites of hydroxylated and polyvinylidene fluoride (PVDF/GROH), which they attributed to strong bonding between the filler and the polymer matrix, where the hydroxyl group acts as a donor. They observed a higher dielectric constant and a lower dielectric loss. A recent study by Wang et al.^[^ ^8^ ^]^ reveals a fourfold improvement of discharging energy density when the polymer, polyvinylidene fluoride (PVDF), is combined with 2D MoS_2_ grown on the surface of 2D transition metal carbide Ti_3_C_2_ MXene (MoS2@MXene). The discharging energy density increased from 3.83 J cm^−3^ for the pure polymer to 17.22 J cm^−3^ for the composite and possess a high dielectric constant and low dielectric loss, 145.08 and 0.06 at 1 kHz, respectively. They attributed these observations to a large specific surface area, high conductivity of MoS_2_@MXene, and its compatibility with the polymer.

There have been numerous studies investigating the use of polymer and 2D nanofiller composites for energy storage applications from flexible supercapacitors,^[^ ^9^, ^10^ ^]^ which are used for storing and releasing energy on demand, to lithium‐ion batteries and hydrogen fuel cells, which have also been shown to benefit from the incorporation of 2D nanofillers.^[^ ^11^, ^12^, ^13^ ^]^ Pan et al.^[^ ^11^ ^]^ studied the performance of MXene‐based nanocomposite polymer electrolytes and reported a substantial improvement in ionic conductivity (2.2 × 10^−5^ S m^−1^ at 28 °C), which they attributed primarily to the 2D geometry of the nanofillers and their good dispersion in the polymer matrix.

**Figure 1:**
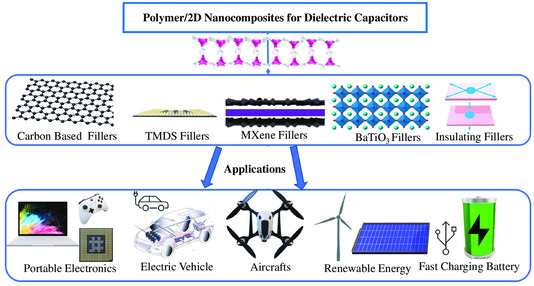
Applications of energy storage devices of polymer nanocomposites.

Despite the promising future of polymer and 2D nanofiller composites for high‐energy‐density energy storage devices and related applications, they inherit several challenges that need to be addressed. First, their stability and durability over long periods, and their performance under severe conditions have not been well assessed. However, early studies suggest that 2D materials improve mechanical properties, thermal stability, and conductivity. For example, Wang et al.^[^ ^14^ ^]^ reported that 2D graphene nanosheets embedded in polyurethane (PU) greatly improve the tensile strength (239%) and storage modulus (202%) of the polymer. Second, defect‐free 2D nanofillers are typically expensive to produce in large quantities, which increase the cost of production of composites. However, a proper combination of polymers and 2D nanofillers has the potential for low‐cost and sustainable energy storage solutions. For example, the polymer matrix can be made from renewable and inexpensive resources such as biomass, while 2D nanofillers such as graphene can be extracted through low‐cost routes.^[^ ^15^, ^16^, ^17^ ^]^ Guardia et al.^[^ ^15^ ^]^ reported a high capacitance of 260 F g^−1^ for a supercapacitor based on biomass wasted carbon and reduced graphene oxide composites. In another study, Nguyen et al.^[^ ^16^ ^]^ observed a high capacitance of 278 F g^−1^ for a composite of biomass carbon dots, derived from cauliflower waste, and reduced graphene oxide. These studies suggest that the overall cost of energy storage systems can be greatly reduced by replacing traditional 2D materials such as graphene and MoS_2_ with alternative materials to lower the cost and reduce the impact on the environment. As research and development efforts continue to advance, we will likely see increased adoption of these materials in the coming years for a wide range of applications, including portable electronics, electric vehicles, and grid‐level energy storage systems. Various applications of polymer‐based energy storage devices are shown in the schematic of **Figure** **1**.

## 2 Demand for High‐Energy‐Density and High‐Power‐Density Devices

**Figure 2:**
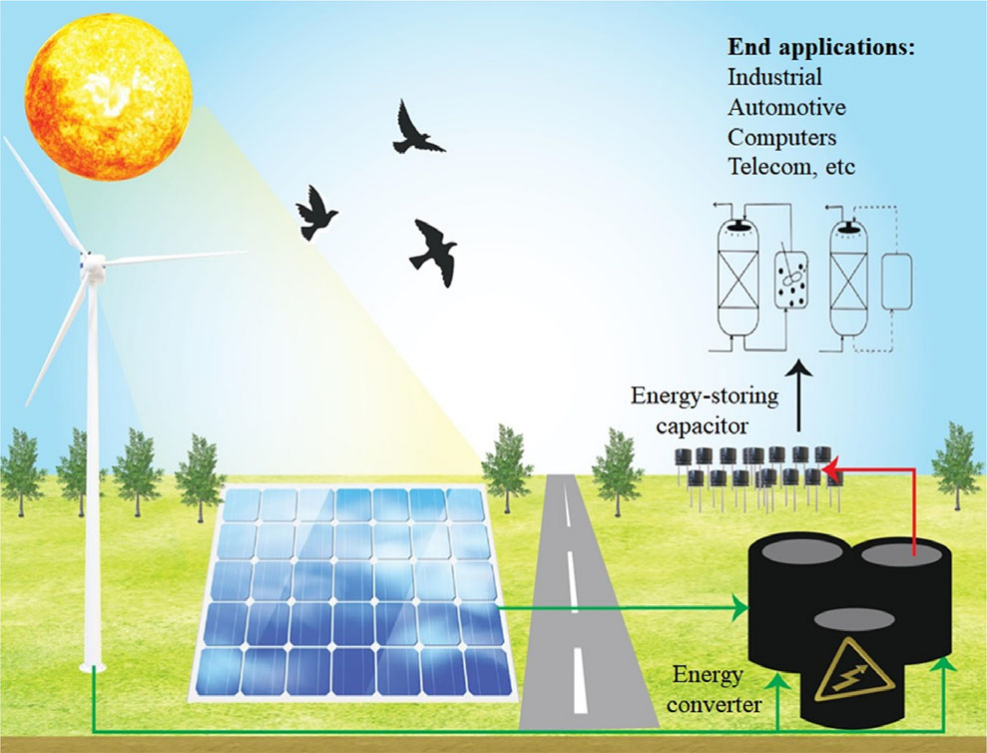
Renewable energy storage mechanism using capacitor batteries.

The demand for energy is booming exponentially with the development of technology and its adoption in daily life which leads toward a rapid depletion of fossil fuels and an energy crisis in the future. Environmentalists have been promoting renewable energy technologies (RET) to minimize the dependency on fossil fuels to protect the planet from global warming and its catastrophic effects.^[^ ^18^, ^19^ ^]^ Despite having endless renewable energy sources and production capabilities, RET faces a major technical barrier due to a lack of meaningful energy storage devices that can store large amounts of energy for a long period of time in a confined space. Although lithium ion‐based storage devices technology was able to overcome this barrier to an acceptable level, they inherit major obstacles such as overheating, slow charging‐discharging, high flammability, and short life cycle that hinder their application in RET. Conversely, capacitor‐based storage devices have fast charging and discharging speeds, and are nonflammable, lightweight, inexpensive, and environmentally friendly. Further, they inherit a longer life cycle compared to conventional energy storage devices that are made of rechargeable lithium‐ion and zinc‐ion batteries. This makes dielectric capacitors‐based storage devices an attractive alternative.^[^ ^20^ ^]^ **Figure** **2** illustrates the process of storing renewable energy with capacitor‐based batteries.

Capacitor‐based devices store energy in the form of charge which gets accumulated on plates of capacitors when connected to a power source within a short period of time. Upon disconnecting from the source and connecting to a load, the stored energy is rapidly released by creating a large current. This fast charging and discharging behavior of capacitors is useful to make pulsed power storage systems that are used in various modern electrical and electronic devices including electric vehicles, weapons, medical devices, and high‐voltage power transmission equipment.^[^ ^20^, ^21^ ^]^

In addition to the electronic industry, capacitors are widely used in packaging, aerospace, biochemical membrane separation, automotive, energy conversion, and electromagnetic interference shielding. However, their low energy densities (*U*_e_) and low threshold temperatures are major barriers that limit their usage for a wide range of applications.

The energy stored in a capacitor is governed by the polarization of the dielectric material under an electric field and the electric field applied. The charged energy density of a dielectric material can be expressed as

(1) $$U=\int_{0}^{D_{\text{max}}}E\text{ d}D$$

where *U* is the charge energy density, *E* is the applied electric field, *D* is the electric displacement, and *D*_max_ is the maximum electric displacement.

(2) $$U_{d}=\int_{D_{r}}^{D_{\text{max}}}E\text{ d}D$$

where *D*_r_ is the residual displacement.

The energy storage efficiency is dictated by the ratio of discharge versus charge energy densities as

(3) $$\eta \;\left(\%\right)=\;\frac{U_{d}}{U}\times 100$$

For linear dielectrics, the electric displacement is proportional to the electric field such that

(4) $$D=\varepsilon_{o}\varepsilon_{r}E$$

where *ε*_o_ is the permittivity of the free space and *ε*_r_ is the relative permittivity of the material. Thus, the energy stored inside the dielectric becomes

(5) $$U=\;\frac{1}{2}\;\varepsilon_{o}\varepsilon_{r}E^{2}$$

The maximum energy density of the material is governed by the maximum electric field that a material can withstand. This maximum electric field is otherwise known as the dielectric breakdown strength of the material (*E*_b_). The maximum energy density of the dielectric (*U*_max_) can then be defined as

(6) $$U_{\text{max}}=\;\frac{1}{2}\;\varepsilon_{o}\varepsilon_{r}E_{b}^{2}$$

Polymers usually have high dielectric strength and low permittivity. Hence, they are considered low‐K materials with high breakdown fields. For example, with a very low dielectric constant and limiting temperature tolerance of biaxially oriented polypropylene (BOPP) based capacitors show a maximum energy density (*U*_e_) of 0.27 J cm^−3^ at 120 °C. In addition, the charge–discharge efficiency of polymer dielectric films rapidly decreases under high electric fields and high temperatures due to an increase in leakage current.^[^ ^22^ ^]^ On contrary, ceramic‐based capacitors have relatively low breakdown voltages as a result of their high permittivity. Inorganic nanoparticles are typically added to polymers to increase their permittivity. However, this decreases the dielectric strength in nanocomposites, thus resulting in a trade‐off between permittivity and dielectric strength.

2D nanosheets, in contrast, can simultaneously increase the permittivity as well the dielectric strength of the polymer nanocomposites. The increased permittivity stems from the surface dipoles, high nanosheet surface area, and synergistic interactions between matrix and nanosheets. The dielectric strength of the nanocomposites based on 2D nanosheets increases due to the “electrical tree” blocking effect of the nanosheets. This causes a substantial improvement in the energy density compared to that of their pure polymer counterparts. Furthermore, 2D nanosheets can have superior mechanical and thermal properties, thus resulting in stronger and thermally stable nanocomposites.

## 3 Advantages of Polymeric Nanocomposites for Dielectric Capacitors

The exponential rise in the usage of electronic devices over the past decade has increased the demand for capacitors. Polymers and ceramics are the most widely used dielectric materials for capacitors. Although the choice between polymer and ceramic dielectric capacitors ultimately depends on the specific requirements of the application, polymer dielectrics perform better than ceramic dielectrics in equivalent series resistance (ESR), flexibility, leakage current, and higher breakdown voltages. These properties make polymer dielectric capacitors more attractive for high‐energy‐density storage devices, although their dielectric constants still need to be improved.^[^ ^23^ ^]^ For more than a decade, many attempts have been made to enhance the thermal stability and the energy density of polymer dielectric capacitors. The incorporation of various types of filler materials is the most efficient way to fulfill the criteria. Owing to their improved thermal and mechanical stability, scalability, and high surface‐to‐volume ratio, 2D fillers are more effective than other filler materials. The addition of 2D nanofillers such as hexagonal boron nitride, mica, graphene oxide, and MXene, etc. improves the holding capacity of charge carriers and impedes the transport of charge carriers. This effectively brings down the dielectric loss up to a certain level while increasing the energy density of the device.^[^ ^24^ ^]^ The major advantages of 2D nanofillers are higher energy densities and improved thermal stability,

### 3.1 High Energy Density

The energy density of a dielectric capacitor primarily depends on two parameters, which are the dielectric constant (*k*) and the breakdown strength. The dielectric constant of a material is a measure of its ability to store electrical charges under the influence of an applied electric field, hence a higher dielectric constant reflects a higher charge storage capacity. Long atomic chains of polymers are held together by weak van der Waals forces. This makes polymers more flexible, easily deformable, and movable, which creates voids within the polymer. These void spaces reduce the charge storage capacity and subsequently the dielectric constant. Moreover, the presence of voids inside the polymer degrades its physical and mechanical properties, making it unstable at high temperatures. 2D fillers such as MXene, mica, hexagonal boron nitride (h‐BN), and graphene fill the free space inside the polymer and enhance the stability and promote the charge occupancy of the polymer matrix, resulting in enhanced dielectric properties.^[^ ^8^, ^25^, ^26^ ^]^ The breakdown strength of a capacitor is the maximum electric field that the capacitor can withstand before it starts leaking charge carriers. The presence of 2D nanofillers inside the polymer impedes the mobility of charges in the direction of the applied electric field which leads to the enhancement of breakdown strength (*E*_b_) of the polymer nanocomposite.^[^ ^23^ ^]^ The improvement of both the dielectric constant (*k*), and the breakdown field (*E*_b_) by incorporating 2D nanofillers into polymer leads to the enhancement of energy density.

### 3.2 High‐Temperature Stability

The operation of energy storage devices based on dielectric capacitors at higher temperatures has been a major issue because polymers start to degrade as the temperature rises near their glass transition temperature. This degradation increases the charge leakage that reduces both the efficiency and the energy density of the device, making it unusable in certain high‐power applications and machinery such as aircraft, hybrid electric vehicles, turbine engines, and many others. Certain polymers such as polyvinylidene fluoride (PVDF), polyetherimide (PEI), and cross‐linked divinyltetramethyldisiloxane‐bis (benzocyclobutene) (c‐BCB)‐based polymers show high‐temperature stabilities up to 150 °C, and incorporation of suitable 2D nanofillers into these polymers improves their sustainability and energy density even at temperatures as high as 200 °C.^[^ ^27^, ^28^, ^29^ ^]^ Moreover, 2D fillers reduce the dielectric loss and improve the efficiency of the capacitor.

## 4 Polymer 2D Nanocomposites

In the past decade, a variety of nanofillers with 3D, 2D, 1D, and 0D have been explored in the area of polymer–nanofiller‐based dielectric capacitors. Polymer–nanofiller composites with 0D and 1D show remarkable performance on dielectric and energy storage.^[^ ^30^, ^31^ ^]^ However, here we have focused on 2D nanofiller‐based dielectric capacitors with materials such as graphene, mica, MXene, boron nitride (BN), MoS_2_, and some 2D oxides. 2D nanofillers have several advantages that can improve the overall performance of the polymer nanocomposite compared to 0D, 1D, or 3D nanofillers. They have a high surface area to volume ratio compared to nanofillers of other dimensions, which promotes a large interfacial area for polymer–nanofillers interactions to increase the interface polarization effect. Thus, a large enhancement of dielectric constants can be expected with low nanofiller contents compared to that of 0D, 1D, or 3D nanofillers.^[^ ^26^ ^]^ Although 0D and 1D nanofillers have the potential of enhancing dielectric properties and energy densities, the chances of agglomeration of fillers increase with increasing the filler content that limits the energy density. Also, studies show that some 2D nanofillers such as h‐BN, graphene, and reduced graphene oxide can greatly improve the thermal stability of the composites in addition to enhancing dielectric properties.^[^ ^32^, ^33^, ^34^ ^]^

### 4.1 Conducting 2D‐Nanofiller‐Based Polymer Dielectric Capacitors

Carbon‐based 2D materials such as carbon nanotube (CNT), graphene, and byproducts of graphene, including graphene oxide (GO) and reduced graphene oxide (rGO), and newly discovered MXene are a class 2D materials with high in‐plane conductivities. As 2D nanofillers, these materials have been extensively studied for their potential role to improve the performance of polymeric dielectric capacitors, owing to their superior physical, electric, thermal, and mechanical properties.

#### 4.1.1 Graphene

Graphene is a semimetal and has superior electrical conduction and charge transport properties which enhance the mobility of charge carriers and the transfer of charges in the polymer matrix, depending on the polymer chain mobility. The permittivity of polymer/graphene composites shows a sudden increase in dielectric behavior after a certain threshold of graphene loading in the polymer by following the percolation theory.^[^ ^35^ ^]^

**Figure 3:**
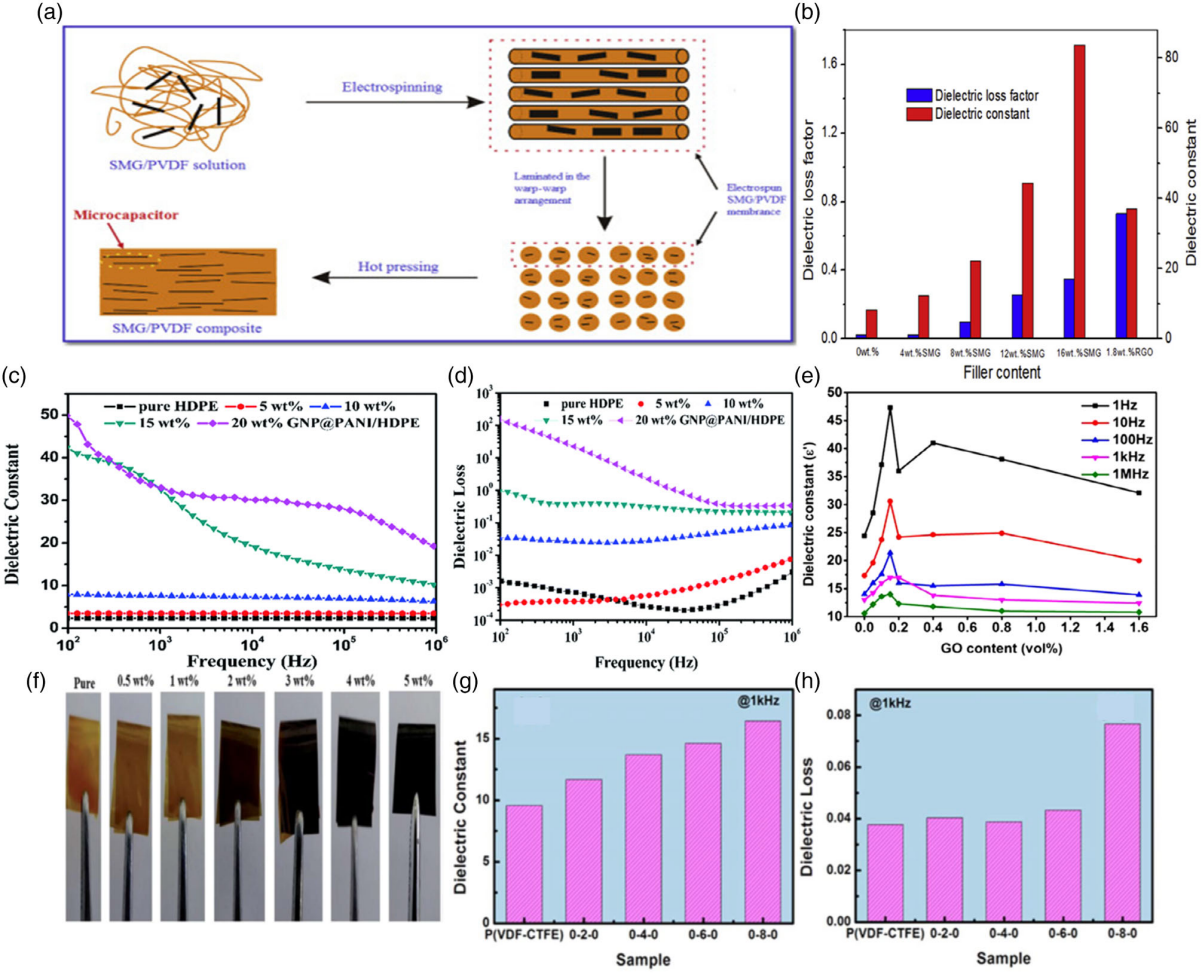
a) Schematic diagram of the fabrication of surface‐modified graphene (SMG)/PVDF composites by an electrospinning‐hot pressing method. Reproduced with permission.^[^ ^36^ ^]^ Copyright 2019, Elsevier. b) Permittivity and dielectric loss factor at 1000 Hz for pristine PVDF and its composites at different wt%. Reproduced with permission.^[^ ^36^ ^]^ Copyright 2019, Elsevier. c,d) Frequency‐dependent electric constant and dielectric loss of pure HDPE and GNP@PANI/HDPE composites at different wt% of GNP@PANI. Reproduced with permission.^[^ ^37^ ^]^ Copyright 2017, Royal Society of Chemistry. e) Variation of the dielectric constant of PVDF/GO with the variation of GO vol% g at different frequencies. Reproduced with permission.^[^ ^40^ ^]^ Copyright 2020, Society of Plastics Engineers, published by Wiley. f) Images of PPD–CFGO/PI composite films. Reproduced with permission.^[^ ^41^ ^]^ Copyright 2015, Royal Society of Chemistry. g,h) The dielectric constant and dielectric loss of pure P(VDF‐CTFE) sandwich‐structured composites at 1 kHz. Reproduced with permission.^[^ ^43^ ^]^ Copyright 2021, IOP Science.

Lin et al. fabricated surface‐modified graphene (SMG)/PVDF films (**Figure** **3**a) by an electrospinning‐hot pressing method which show a tremendous enhancement of dielectric properties with a dielectric constant as high as 83.8 at a loading of 16 vol% of SMG with a relatively low dielectric loss factor (0.34), as shown in Figure 3b.^[^ ^36^ ^]^ Also, Figure 3c,d shows the largest enhancement of the dielectric constant in GNP/PANI dispersion inside HDPE polymer from 2 to 50 with 20% GNP/PANI loading.^[^ ^37^ ^]^ As shown in Figure 3d, a huge dielectric loss was measured from 10^−3^ to 10^2^ as a function of increased GNP/PANI filler concentration. Xu et al. added graphene and ionic liquid‐modified graphene (GIL) 2D fillers into PVDF polymer and prepared PVDF/GIL and PVDF/graphene polymer nanocomposites by solution‐cast method.^[^ ^38^ ^]^ They observed a higher dielectric permittivity with low dielectric loss in the PVDF/GIL composites; however, the observed permittivity is lower, and the loss is higher, contrary to what's observed for PVDF/graphene composites. These nanocomposites showed percolation thresholds, approximately 1.86 vol% for PVDF/GIL and 0.67 vol% for PVDF/Graphene films.^[^ ^38^ ^]^ In another study, Lv et al.^[^ ^39^ ^]^ deposited polyaniline (PANI) on exfoliated graphite nanoplates (*x*GNPs) grown by in situ polymerization method, to fabricate PANI‐coated xGNPs (*x*GNPs@PANI) conductive fillers for the oxidized styrene–butadiene–styrene copolymer (SBS‐FH), which contains both hydroxyl and formyloxy groups. The *x*GNPs@PANI/SBS‐FH composite exhibited a dielectric constant of 56.8 and a dielectric loss factor of 0.51 at 1000 Hz near the percolation threshold filled with just 9.38 vol% of *x*GNPs@PANI. The corresponding values of *x*GNPs (1.19 vol%)/SBS composite were 15.96 and 2.91 at 1000 Hz, respectively.^[^ ^39^ ^]^ Although the incorporation of graphene in polymer matrices improves the dielectric constant by multiple orders, its high electrical conductivity causes high dielectric losses, making graphene an unfitting candidate for high energy storage applications unless a suitable method is applied to prevent the loss.^[^ ^4^ ^]^

#### 4.1.2 Graphene Oxide

Muduli et al. utilized solution casting followed by a hot press technique to prepare PVDF/GO films.^[^ ^40^ ^]^ They observed an enhancement in dielectric constants with increasing GO loading, up to 0.15 vol%, but a further increase of GO caused a decrease in dielectric constant (Figure 3e). As the GO content in the polymer increases, the remnant polarization increases whereas energy density followed a similar trend as the dielectric constant. Fang et al. synthesized NH_2_‐functionalized and carboxyl‐functionalized graphene oxide (PPD–CFGO)/polyimide (PI) composites shown in Figure 3f, which exhibited an enhancement of dielectric constants up to 36.9 (12.5 times the pure polymer) while maintaining the dielectric loss to extremely low values (≈0.0075).^[^ ^41^ ^]^ GO is a derivative of graphene, which is less conductive than graphene due to the presence of oxygen‐containing functional groups. In addition, the oxygen groups in GO improve the dielectric properties of the polymer nanocomposites by enhancing the dielectric constant with relatively low tangent loss.^[^ ^42^ ^]^

Cheng et al. incorporated GO into poly(vinylidene fluoride‐*co*‐chlorotrifluoroethylene) (P(VDF‐CTFE)) to prepare GO/P(VDF‐CTFE) film by casting method. The composite with 0.4 wt% of GO nanosheets exhibited a dielectric constant of 13.6 (at 1 kHz) with a higher discharged energy density of 8.25 J cm^−3^ at an applied field of 3,400 kV cm^−1^ (Figure 3g,h).^[^ ^43^ ^]^ In another report, Rathod et al. fabricated composite films of poly(vinyl alcohol) (PVA) doped with functionalized GO by solution casting method. Although the films showed ultra‐high dielectric constants up to 400 with 10 wt% of GO, the dielectric loss was higher (≈6).^[^ ^44^ ^]^ As a result of this high tangential loss which increases with the loading percentage, GO nanofillers are less likely to be incorporated into high‐power‐density dielectric polymer capacitors.

#### 4.1.3 Reduced Graphene Oxide (rGO)

**Figure 4:**
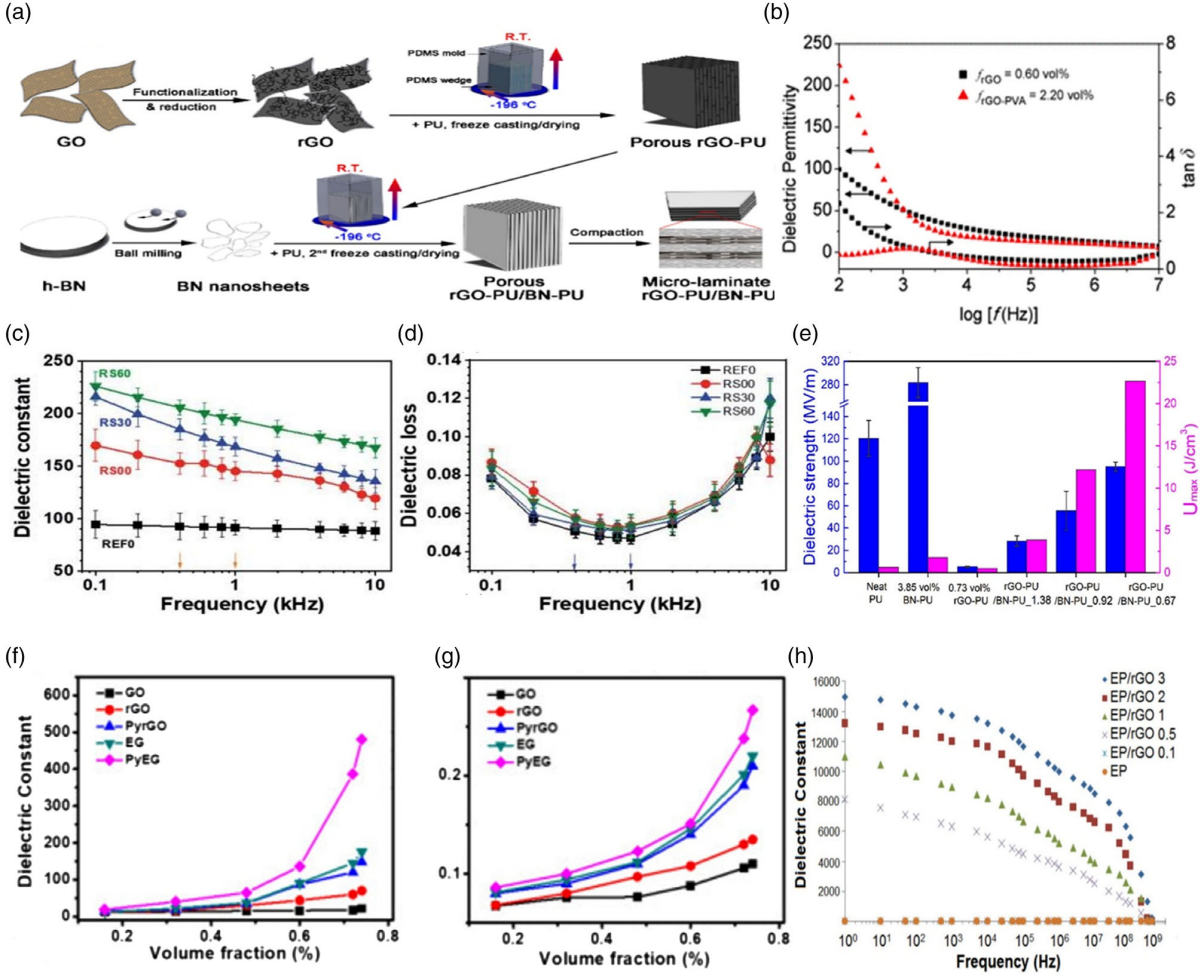
a) Schematics of the synthesis of highly aligned rGO–PU/BN–PU microlaminate composites. Reproduced with permission.^[^ ^47^ ^]^ Copyright 2018, American Chemical Society. b) Dielectric permittivity and loss of rGO/PVDF and rGO‐PVA/PVDF nanocomposites at a filler volume fraction near the percolation threshold. Reproduced with permission.^[^ ^45^ ^]^ Copyright 2012, American Chemical Society. c,d) Frequency‐dependent dielectric constant and dielectric loss of BTO and rGO@BTO films. Reproduced with permission.^[^ ^46^ ^]^ Copyright 2022, Royal Society of Chemistry. e) Dielectric strength and energy density of PU, rGO‐PU, BN‐PU, and rGO‐PU/BN‐PU Composites. Reproduced with permission.^[^ ^47^ ^]^ Copyright 2018, American Chemical Society. f–g) The dielectric constants and losses of GO/PVDF, rGO/PVDF, PyrGO/PVDF, EG/PVDF, and PyEG/PVDF composites with different filler contents. Reproduced with permission.^[^ ^50^ ^]^ Copyright 2019, Elsevier. h) Dielectric constants of rGO/epoxy nanocomposites as a function of frequency. Reproduced with permission.^[^ ^51^ ^]^ Copyright 2014, Wiley‐VCH.

Reduced graphene oxide (rGO) is a 2D material that is similar to graphene which is produced by reducing GO. Owing to its high surface area, good compatibility with polymers, and biodegradable nature, rGO can be considered as a preferred 2D nanofiller for the fabrication of polymer nanocomposite capacitors for high‐energy storage applications. Wu et al. followed a two‐step bidirectional freeze‐casting process to synthesize microlaminate composites consisting of alternating reduced graphene oxide (rGO) and h‐BN nanosheets embedded in a polyurethane (PU) matrix as shown in **Figure** **4**a. Further, Wang et al. observed a huge enhancement of dielectric permittivity of the nanocomposites near the percolation thresholds which were 2.24 vol% for rGO‐PVA/PVDF and 0.61 vol% for rGO/PVDF composites, respectively, as shown in Figure 4b.^[^ ^45^ ^]^ Jun et al. fabricated rGO‐encapsulated BTO (rGO@BTO) films that showed an increase in dielectric constants up to 194 with a low dielectric loss of 0.053 due to the interfacial polarization,^[^ ^46^ ^]^ as shown in Figure 4c,d. The rGO−PU/BN−PU microlaminate composites exhibited dielectric constants as high as 1084 with a low dielectric loss of 0.091 at 1 kHz and a maximum energy density of 22.7 J cm^−1^,^[^ ^3^, ^47^ ^]^ as shown in Figure 4e. This is due to h‐BN's high dielectric constant (*k*) which can enhance the breakdown voltage substantially. Yuan et al. reported rGO/PDMS composites that exhibited unprecedented dielectric properties with high dielectric constants up to 753 at 100 Hz and low loss tangents of 0.4.^[^ ^48^ ^]^ Almafie et al. prepared polyacrylonitrile (PAN) and reduced graphene oxide nanocomposites (PAN/GO) by electrospinning. Although they obtained a high dielectric constant of 86.4 at 102 Hz, the dielectric loss has enhanced to 4.97.^[^ ^49^ ^]^ Li et al. incorporated exfoliated graphene (EG), graphene oxide (GO), and reduced graphene oxide (r‐GO) separately into PVDF through 3,4,9,10‐perylenetetracarboxylic acid (*P*_*y*_) to prepare GO/PVDF, rGO/PVDF, P_*y*_rGO/PVDF, EG/PVDF, and P_*y*_EG/PVDF composites with different filler contents. The P_*y*_EG/PVDF showed the highest enhancement of dielectric constant as shown in Figure 4f,g, up to 480 with 0.74 vol% of EG with a comparatively low dielectric loss of 0.27.^[^ ^50^ ^]^ The study by Yousef et al. reported dielectric constants of over 14 000 with 3 wt% of rGO/lyotropic nematic liquid crystal to form rGO/epoxy composite, leading to its potential usage in electromagnetic interference shielding devices^[^ ^51^ ^]^ which is shown in Figure 4h.

Wang et al. incorporated rGO and poly(vinyl alcohol) (PVA)‐modified rGO (rGO‐PVA) into PVDF to prepare rGO/PVDF and rGO‐PVA/PVDF composites films by solution‐cast method and explored their dielectric properties. An insulator‐to‐conductor percolating transition was observed for both composites as the filler content was increased, which was explained by the interfacial polarization effect and the microcapacitor model.^[^ ^45^ ^]^ A similar observation of high dielectric constants with moderate dielectric losses in MS (melamine sponge)/rGB/silicone rubber (SR) composites was reported by Zhang et al.^[^ ^52^ ^]^ Yaqoob et al. investigated the impact of the presence of rGO in dielectric PVDF‐BTO films. They reported a high dielectric constant and an energy density of 98 and 4.5 J cm^−3^, respectively with a relatively low loss of 0.081 at 1 MHz for the PVDF‐BTO‐RGO 0.3 wt% sample.^[^ ^53^ ^]^ There are other studies conducted to explore dielectric properties of rGO/polymer nanocomposites; however, their reported efficiencies were comparably low.^[^ ^45^, ^46^, ^48^, ^49^, ^50^, ^51^, ^52^, ^53^, ^54^, ^55^, ^56^ ^]^

#### 4.1.4 MXene

**Figure 5:**
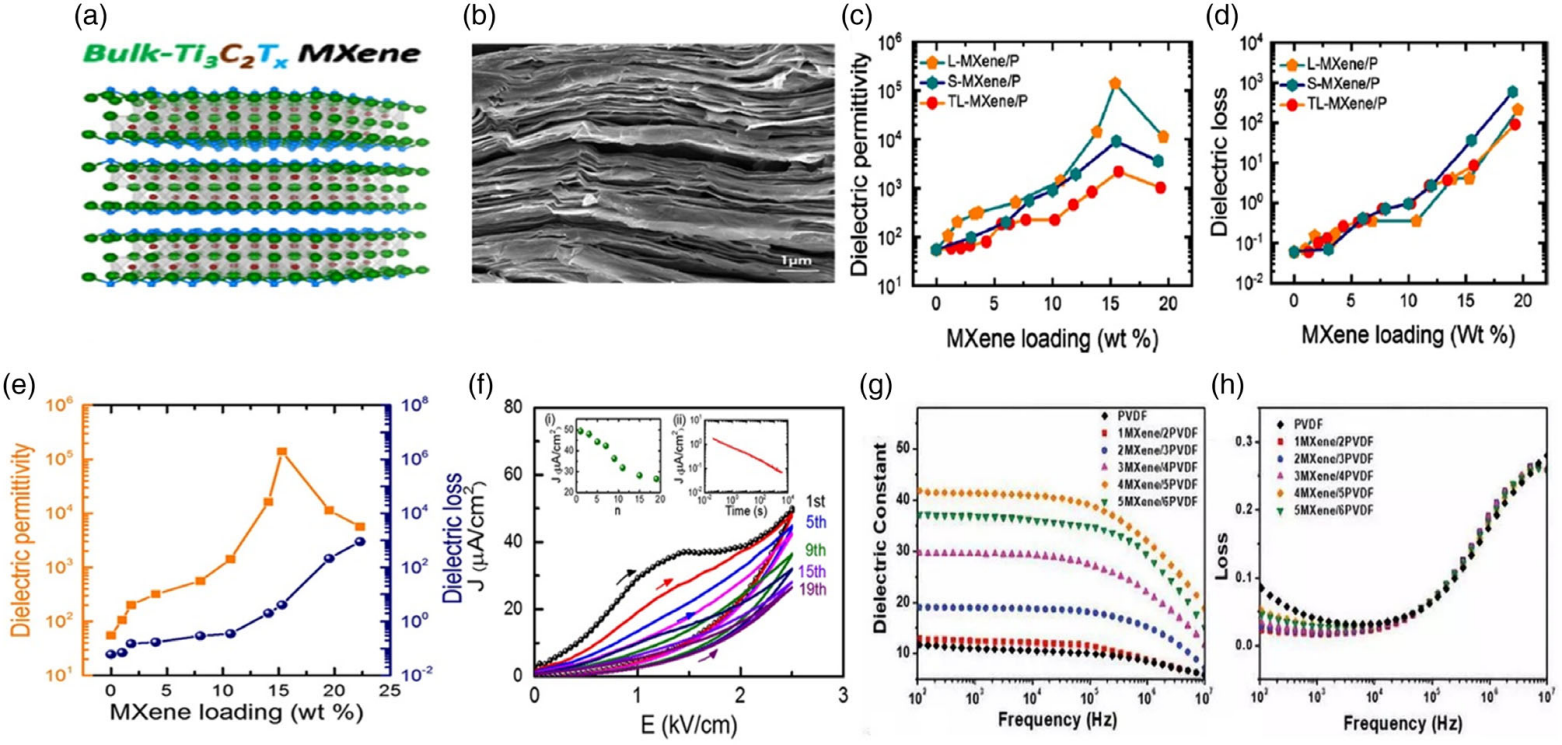
a) Schematics of bulk‐Ti_3_C_2_T_*x*_ MXene structure. Reproduced with permission.^[^ ^26^ ^]^ Copyright 2018, American Chemical Society. b) Cross‐sectional SEM image. Reproduced with permission.^[^ ^58^ ^]^ Copyright 2019, American Chemical Society. c,d) Dielectric permittivity and dielectric loss of the LMXene/P(VDF‐TrFE‐CFE), S‐MXene/P(VDF‐TrFE‐CFE), and TLMXene/ P(VDF‐TrFE‐CFE) composites versus MXene loading measured at room temperature and 1 kHz. Reproduced with permission.^[^ ^58^ ^]^ Copyright 2019, American Chemical Society. e) The permittivity and dielectric loss of MXene/P(VDF‐TrFE‐CFE) at room temperature and 1 kHz as a function of MXene wt%. Reproduced with permission.^[^ ^26^ ^]^ Copyright 2018, American Chemical Society. f) Unipolar *J–E* loops of the MXene/P(VDF‐TrFE‐CFE) composites (8.0 wt% MXene), measured with a positive field applied after being prepoled with a negative field of 2.5 kV cm^−1^. Reproduced with permission.^[^ ^58^ ^]^ Copyright 2019, American Chemical Society. The inset (i) shows the decrease in current density at the maximum field with the number of cycles of the field. The inset (ii) illustrates the time‐dependent leakage current density measured under a constant field of 1.25 kV cm^−1^ for 1 h. Reproduced with permission.^[^ ^61^ ^]^ Copyright 2019, Royal Society of Chemistry. g,h) Variation of the dielectric constant and dielectric loss with the frequency for MXene/PVDF films with a different number of MXene layers. g,h) Reproduced with permission.^[^ ^61^ ^]^ Copyright 2019, Royal Society of Chemistry.

MXene is a 2D layered material that is composed of transition metal carbides or nitrides, and it has been shown to improve the dielectric properties of polymer matrix composites (**Figure** **5**a,b). MXene can be added to the polymer matrix in the form of flakes, which can act as an effective filler material. The high surface area of MXene flakes increases the total interfacial area between the MXene and the polymer, which can lead to improved electrical conductivity and a reduction in dielectric constants of the composite material. MXene also has a high thermal conductivity, which can help to dissipate heat from the polymer matrix and further improve its dielectric properties. In 2014, in the first report of MXene being used as a 2D filler in polymers, Ti_3_C_2_T_*x*_ MXene was added as nanofillers to polydiallyldimethylammonium chloride (PDDA) and PVA to study various properties such as electrical conductivity, the capacitive performance, and mechanical strength.^[^ ^57^ ^]^ Since then, the research of MXene–polymer nanocomposites (NCs) gained significant attention and interest for similar studies with various polymers. Recently, Tu et al. reported that PVDF‐based composites with 2D MXene nanosheets as fillers have significantly enhanced dielectric permittivity.^[^ ^26^ ^]^

Using P[VDF‐TrFE‐CFE] polymer with Ti_3_C_2_T_*x*_ nanosheets, dielectric permittivity reaches 10^5^ for 15.0 wt% MXene loading, surpassing previous carbon‐based composites (Figure 5c).^[^ ^26^, ^58^ ^]^ At 10 wt% loading of MXene, the dielectric constant made a 25‐fold increase for a fivefold increase in dielectric loss from 0.06 to 0.35, as shown in Figure 5d. Further, MXene/polymer composites have a superior permittivity‐to‐loss ratio compared to other 2D fillers, as shown in Figure 5e. The dielectric constant enhancement has been observed in other polymers with MXene loading, caused by charge accumulation from microscopic dipoles at MXene/polymer interfaces under an electric field.^[^ ^26^ ^]^ In another study, they examined the dielectric properties of MXene/polymer composites with surface termination, which shows a significant impact on their dielectric permittivity (Figure 5f).^[^ ^58^ ^]^ Mazhar et al. reported the fabrication of flexible PVC/MXene nanocomposites with high flexibility, increased thermal conductivity (3.48 W m^−1^ K^−1^), extraordinary thermal stability (683.8 °C), and good mechanical stability (tensile strength ≈174.08%), high dielectric constant (11 800) and low energy loss.^[^ ^59^ ^]^ Yu et al. observed that polyimide‐based (PI) nanocomposites containing 2D oxidized MXene Ti_3_C_2_T_*X*_ suppressed leakage current and greatly improved breakdown strength and enhanced capacitive performances at elevated temperatures. They achieved the largest discharged energy density (*U*_e_) of 8.67 J cm^−3^ with an efficiency of 84.1% at 648 kV mm^−1^ with 0.5 wt% oxidized MXenes/PI nanocomposites at room temperature; however, *U*_e_ decreased to 5.46 and 2.05 J cm^−3^ at 100 and 150 °C, respectively.^[^ ^60^ ^]^ Li et al. reported the fabrication of multilayer‐structure nanocomposites comprising four layers of MXene and five layers of poly(vinylidene fluoride) (PVDF) by spin coating, spray coating, and hot‐press methods.^[^ ^61^ ^]^ This multilayer capacitor exhibited a high dielectric constant of 32.2, a maximum discharge energy density of 7.4 J cm^−3^, and a low dielectric loss of 0.5 at 1 MHz, as shown in Figure 5g,h. With exceptional transport properties, MXene will be utilized in the future to make various polymer composite dielectric capacitors that may improve energy densities beyond the current limits.

### 4.2 Semiconducting 2D‐Nanofiller‐Based Polymer Dielectric Capacitors

#### 4.2.1 MoS_2_

The addition of semiconducting 2D nanofillers, such as MoS_2_ and WS_2_ to polymer dielectric capacitors improves dielectric properties of the polymer by several magnitudes,^[^ ^62^, ^63^, ^64^, ^65^, ^66^, ^67^, ^68^, ^69^ ^]^ however, other semiconducting materials other than MoS_2_ have received little attention. The presence of these 2D nanofillers in the polymer matrix also increases the charge storage capacity, leading to an enhancement in energy storage performance. Additionally, they act as barriers that limit the movement of charges and decrease losses, which leads to a decrease in the loss tangent.

**Figure 6:**
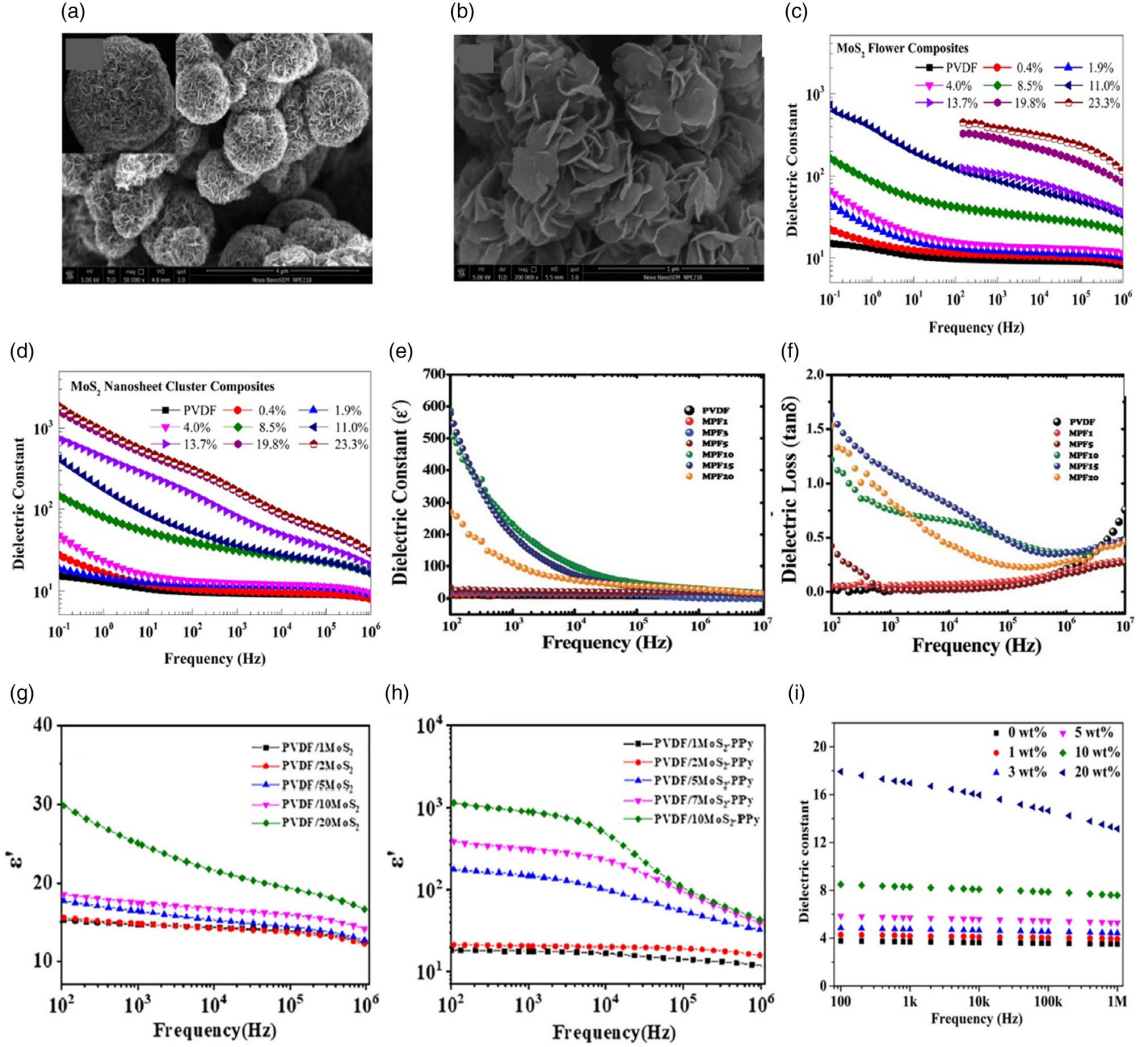
a,b) SEM images of MoS2 hydrangea‐like flowers (a) and MoS_2_ nanosheet clusters (b). c,d) Variation of the dielectric constant of PVDF/MoS_2_ flower composite and PVDF/MoS_2_ nanocluster composite. Reproduced with permission.^[^ ^62^ ^]^ Copyright 2016, American Chemical Society. e,f) Variation of dielectric constant and dielectric loss with frequency and of MoS_2_–PANI/PVDF composite films 25 °C. e,f) Reproduced with permission.^[^ ^69^ ^]^ Copyright 2017, Royal Society of Chemistry. g,h) Variation of the dielectric constant and dielectric loss of PVDF/MoS_2_ and PVDF/MoS_2_–PPy samples. Reproduced with permission.^[^ ^65^ ^]^ Copyright 2020, Elsevier. i) Variation of the dielectric constant of various MoS_2_@(PDA + PEI)/PEN nanocomposite films. Reproduced with permission.^[^ ^68^ ^]^ Copyright 2021, Elsevier.

MoS_2_ has been used as a nanofiller mostly with poly(vinylidene fluoride) (PVDF).^[^ ^62^, ^63^, ^64^ ^]^ The different morphologies of flower‐like and nanosheet clusters like MoS_2_ synthesized using hydrothermal method, as shown in **Figure** **6**a,b. They observed an enhancement of energy densities up to 4.1 J cm^−3^ at 200 MV m^−1^. This was achieved by introducing 0.4% hydrangea‐like flower MoS_2_ and nanosheet cluster MoS_2_, respectively in PVDF.^[^ ^62^ ^]^ Maity et al. used polyaniline (PANI) as an interlinker polymer between PVDF and MoS_2_ and observed that MoS_2_–PANI/PVDF (MPF) composite film with 10% filler content exhibited a maximum increment of dielectric constant of approximately 586 at 100 Hz, though they were unable to control the dielectric loss, as shown in Figure 6e,f.^[^ ^69^ ^]^ Pan et al. fabricated dielectric composites by combining molybdenum disulfide–polypyrrole (MoS_2_–PPy) hybrids with PVDF that exhibited improved dielectric properties as shown in Figure 6g,h.^[^ ^65^ ^]^ Further, the incorporation of MoS_2_ nanofillers in biodegradable environmentally friendly natural polymers such as chitin and other crosslinking hybrid polymers has been reported (Figure 6i).^[^ ^66^, ^67^ ^]^ Polydopamine (PDA) and polyethyleneimine (PEI) crosslinked into the poly(arylene ether nitrile) (PEN) matrix and mixed with hydrothermally synthesized MoS_2_ to form MoS_2_@(PDA + PEI)/PEN nanocomposites, which showed excellent dielectric performance.^[^ ^66^ ^]^ Also, Feng et al. observed enhanced dielectric constants in PEN/MoS_2_@PDA composite films.^[^ ^67^ ^]^

### 4.3 Insulating 2D‐Nanofiller‐Based Polymer Dielectric Capacitors

Insulating 2D‐nanofiller‐based polymer dielectric capacitors have gained significant attention in recent years due to their unique properties and potential applications. These capacitors use a polymer material as the dielectric layer, which is filled with 2D nanomaterials such as h‐BN, boron nitride nanosheets (BNNS), mica, MMT, Silicates, TiO_2_, Al_2_O_3_, and CNOs. These 2D nanofillers provide unique properties such as high dielectric constant and improved thermal stability, making them suitable for a wide range of applications, from portable electronics to sensor systems and energy storage devices.

#### 4.3.1 Hexagonal Boron Nitride Nanosheets

BNNS is a wide‐bandgap (*E*_g_ = 6 eV) material that has a similar structure to graphene and is widely used as a gate dielectric material in electronic devices of various 2D materials including graphene and transition metal dichalcogenides (MoS_2_, WS_2_, MoTe_2_, etc.)^[^ ^70^, ^71^ ^]^ due to its excellent thermal, electrical conductivity and mechanical strength. BNNS are often used in polymers to suppress leakage current by blocking the carrier transport pathways in the polymer layer, thereby improving dielectric performance by improving *E*_b_ and *η*.

Incorporation of BNNS in various polymers such as PVDF,^[^ ^72^, ^73^, ^74^ ^]^ poly(methyl methacrylate) (PMMA),^[^ ^75^ ^]^ polycarbonate PC,^[^ ^76^ ^]^ polyetherimide (PEI),^[^ ^77^ ^]^ polyimide (PI),^[^ ^78^ ^]^ and polyether ether ketone) (PEEK),^[^ ^79^ ^]^ leads to a substantial improvement of dielectric properties of their nanocomposites.

**Figure 7:**
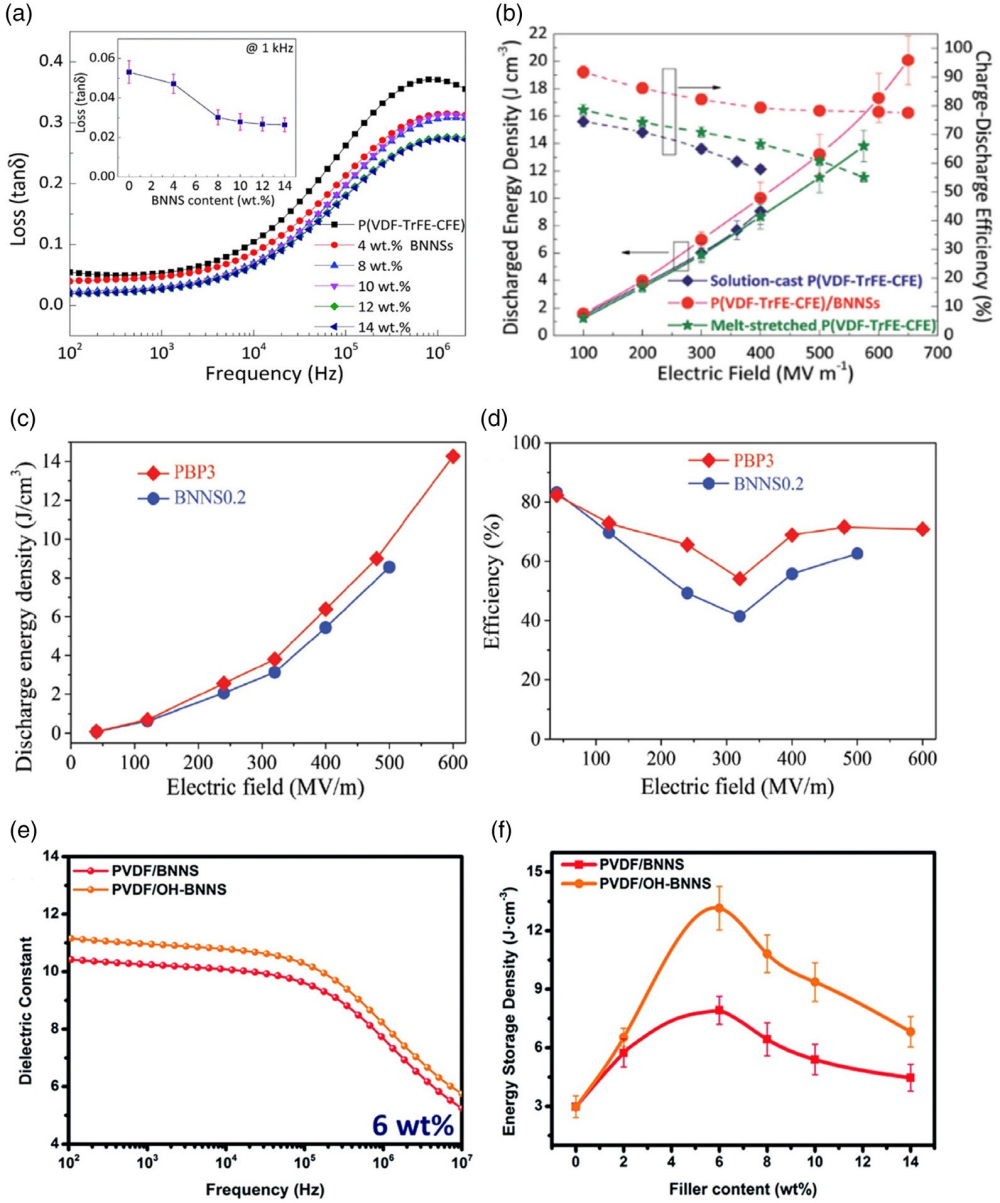
a) Variation of dielectric loss of P(VDF‐TrFECFE)/BNNS nanocomposites with the frequency for different wt% of BNNS. The inset shows the dielectric loss of P(VDF–TrFE–CFE)/BNNS nanocomposites as a function of filler content at 1 kHz. Reproduced with permission.^[^ ^72^ ^]^ Copyright 2015, Royal Society of Chemistry. b) Comparison of the energy density and charge–discharge efficiency of pristine P(VDF–TrFE–CFE) and P(VDF–TrFE–CFE)/BNNS nanocomposites with 12% BNNS at various electric fields. Reproduced with permission.^[^ ^72^ ^]^ Copyright 2015, Royal Society of Chemistry. c,d) Energy density and charge–discharge efficiency of PVDF/BNNS sandwiched structure of different vol% of BNNS. Reproduced with permission.^[^ ^73^ ^]^ Copyright 2019, Wiley‐VCH. e) Variation of the dielectric constant of PVDF/BNNS and PVDF/OH‐BNNS nanocomposites with the frequency with 6 wt% filler content. f) Variation of the energy storage density as functions of BNNSs (OH‐BNNSs) fraction. Reproduced with permission.^[^ ^74^ ^]^ Copyright 2018, Royal Society of Chemistry.

For example, Li et al. showed that utilization of 12 vol% of BNNS in ferroelectric P(VDF‐TrFE‐CFE) terpolymers greatly improves the charge–discharge efficiency up to 83%, and *U*_e_ up to 20.3 J cm^−3^ at 650 MV m^−1^.^[^ ^72^ ^]^ The loss starts increasing above the frequency of 10 000 Hz, as shown in **Figure** **7**a. A study conducted by Zhu et al., for PVDF‐BNNS composite films, revealed that the assimilation of BNNS helps to diminish the local field distortion and propagation of electrical treeing. They observed that the breakdown strength and the energy density enhanced by 136% and 275% compared to pure PVDF, with 0.16 vol% of nanofillers, as shown in Figure 7b.^[^ ^73^ ^]^ The energy density and the efficiency of two such sandwiched polymer nanocomposites with different BNNS wt% are shown in Figure 7c,d.

Surface hydroxylation of BNNS boosts dielectric properties in PVDF/OH‐BNNS resulting in an impressive enhancement in energy density of 13.1 J cm^−3^ with only 6% filler content, as reported by Wu et al.^[^ ^74^ ^]^ Figure 7e,f shows the variation of dielectric constant with frequency and energy density with filler concentration. The addition of BNNS with crosslinked polymers such as poly (aryl ether ketone) (DPAEK) and [divinyltetramethyldisiloxane‐bis(benzocyclobutene)] BCB improved dielectric properties of the composites at high temperature.^[^ ^27^, ^80^ ^]^ It has also reduced dielectric relaxation and improved the breakdown strength and efficiency. Li et al. reported that composites of BNNS (10% vol) and c‐BCB polymer exhibit 90% charge–discharge efficiency (η) at the breakdown strength (*E*_b_) of 400 MV m^−1^ at 150 °C. Even at higher temperatures such as 200 and 250 °C with fields as high as 400 MV m^−1^, the c‐BCB/BNNS composites maintained relatively high η.^[^ ^27^ ^]^

Azizi et al. observed a large improvement in discharged energy densities (up to 1.2 J cm^−3^) and charge–discharge efficiencies (90%) in polyetherimide (PEI) sandwiched with CVD‐grown hexagonal boron nitride (h‐BN) films at 217 °C.^[^ ^80^ ^]^ Liu et al. fabricated polymer nanocomposite capacitors by sandwiching polycarbonate (PC) between two hexagonal boron nitride (h‐BN) layers. The dielectric properties of the h‐BN/PC/h‐BN capacitors vary with the thickness of h‐BN and exhibited a low leakage current density, and a high breakdown strength when the thickness of the h‐BN was 1 μm. They calculated *U*_e_ to be 5.52 J cm^−3^ for the maximum field of 500 MV m^−1^ at 100 °C. As the thickness of the h‐BN increased the dielectric properties started diminishing.^[^ ^76^ ^]^

#### 4.3.2 Mica

Mica is one of the most widely used dielectric materials with high chemical, mechanical, and electrical stability with a bandgap of 7.85 eV and a dielectric constant of 6–9.^[^ ^81^ ^]^ These properties made mica, particularly 2D sheets and nanofillers, an excellent candidate to integrate into polymers to improve both dielectric and mechanical properties. Recent studies by Gao et al. demonstrated that 2D‐mica increases the shear stress and facilitates an ordered formation of polymer chains through strong interactions between the polymer and 2D‐mica fillers at their interfaces.^[^ ^82^ ^]^

**Figure 8:**
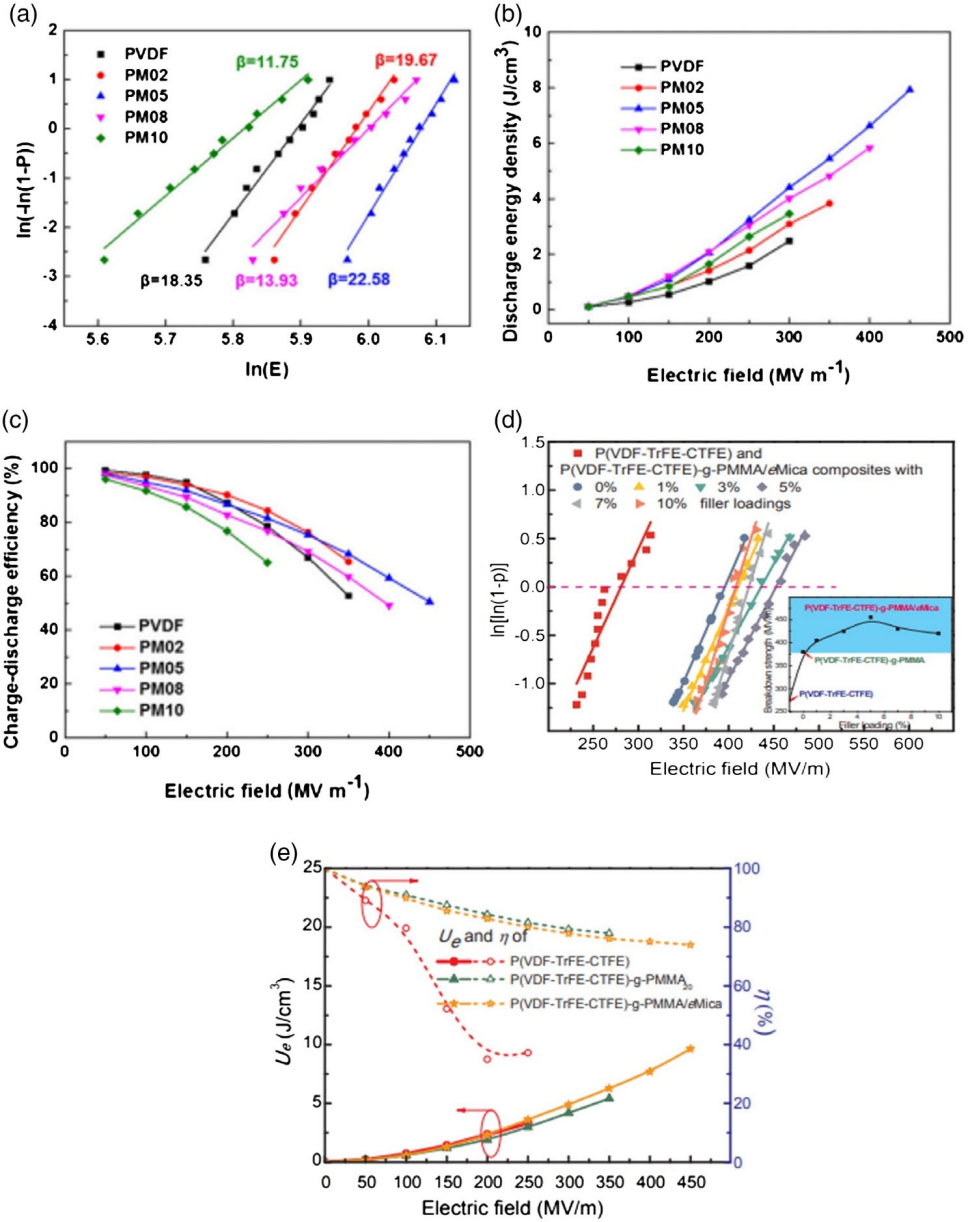
a–c) Variation of the Weibull breakdown strength (a), the discharge energy density (b) and the charge–discharge efficiency (c) with respect to the applied electric field (*E*) for different shape parameters (*β*) for PVDF and PVDF–mica composites with different volume fractions of Mica nanosheets. Reproduced with permission.^[^ ^83^ ^]^ Copyright 2019, Society of Plastics Engineers, published by Wiley. d,e) Variation of Weibull breakdown strength (d) and discharge energy density and charge–discharge efficiency (e) with respect to the applied electric field for P(VDF–TrFE–CTFE)‐g‐PMMA/mica composites with different weight fractions of exfoliated mica. Reproduced with permission.^[^ ^84^ ^]^ Copyright 2019, Elsevier.

A study conducted by Fu et al. shows that the incorporation of mica into PVDF significantly improves its breakdown field (*E*_b_), and discharge energy density without compromising its charge–discharge efficiency,^[^ ^83^ ^]^ as shown in **Figure** **8**a–c. They reported an increase in *E*_b_ from 360 to 450 MV m^−1^ for the composite with 5% mica volume fraction (PM05), which they attributed to decreased mobility and restrained charge transfer due to the strong interaction between the polymer and nanosheets. Also, the composite exhibited improved performance in energy storage capacity with the highest energy density of 7.93 J cm^−3^ for the 5% mica composite (PM05) at 450 MV m^−1^, a 320% improvement compared to the pure PVDF (2.47 J cm^−3^). Further, the composite was able to retain an efficiency above 75% for fields up to 300 MV m^−1^ by exceeding the performance of the pure polymer as a result of low leakage currents due to the insulating nature of mica. Similar behavior for mica–polymer composites has been reported by Wang et al. They incorporated exfoliated mica nanosheets to grafted P(VDF‐TrFE‐CTFE)‐g‐PMMA terpolymer and observed an improvement in *E*_b_, energy density, and charge–discharge efficiency.^[^ ^84^ ^]^ As shown in Figure 8d,e, *E*_b_ increases from 390 to 450 MV m^−1^, and the energy density increased from 5.4 to 9.6 J cm^−3^ for composite samples while maintaining high‐efficiency rates, above 75%, even at 450 MV m^−1^.

**Figure 9:**
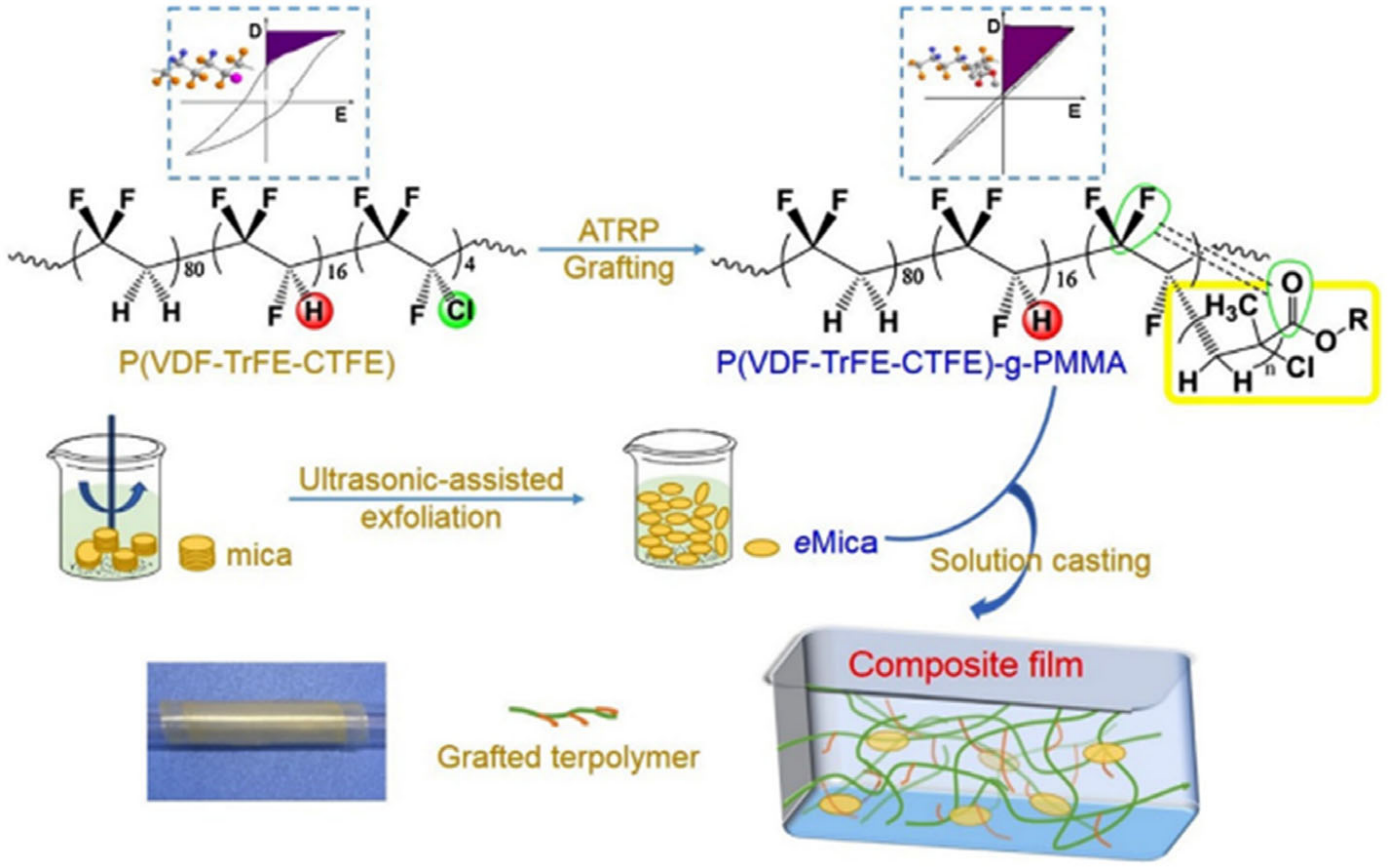
Schematics of the preparation of polymer/exfoliated‐mica nanocomposite film by solution casting. Reproduced with permission.^[^ ^84^ ^]^ Copyright 2019, Elsevier.

Typically, nanocomposites are made by solution casting of exfoliated mica nanosheets and polymer solutions.^[^ ^72^, ^85^, ^86^ ^]^ As shown in **Figure** **9**, exfoliated mica nanosheets are prepared by sonication of mica powder in a volatile solvent such as DMF followed by rapid centrifugation (≈15 000 rpm) to remove large nanosheets, which are then filtered and dried at 70 °C overnight in a reduced vacuum to remove the solvent,^[^ ^83^ ^]^ to get the precipitate of nanosheets with near homogeneous size distribution. These as‐prepared mica nanosheets are dispersed in DMF with different weight fractions and vigorously mixed with the polymer solution and vacuumed to remove any trapped air bubbles. Then the mixture is cast onto preheated glass slides followed by heating at 75–100 °C for several hours to completely remove the solvent.

#### 4.3.3 Aluminum Oxide (Al_2_O_3_)

Aluminum oxide Al_2_O_3_ is a cost‐effective and one of the most abundant ceramics. Li et al.^[^ ^29^ ^]^ demonstrated the creation of polymer nanocomposites made of Al_2_O_3_ fillers with various morphologies (nanoparticles, nanowires, and nanoplates) created via a solution‐based technique. They discovered that the conductivity and the breakdown strength at high temperatures have a strong correlation to the shape of the Al_2_O_3_ fillers. These composites exhibited exceptional capacitive performance with a discharged energy density of 3.31 J cm^−3^ and a charge–discharge efficiency of over 90% when measured at 450 MV m^−1^ at 150 °C.^[^ ^29^ ^]^

**Figure 10:**
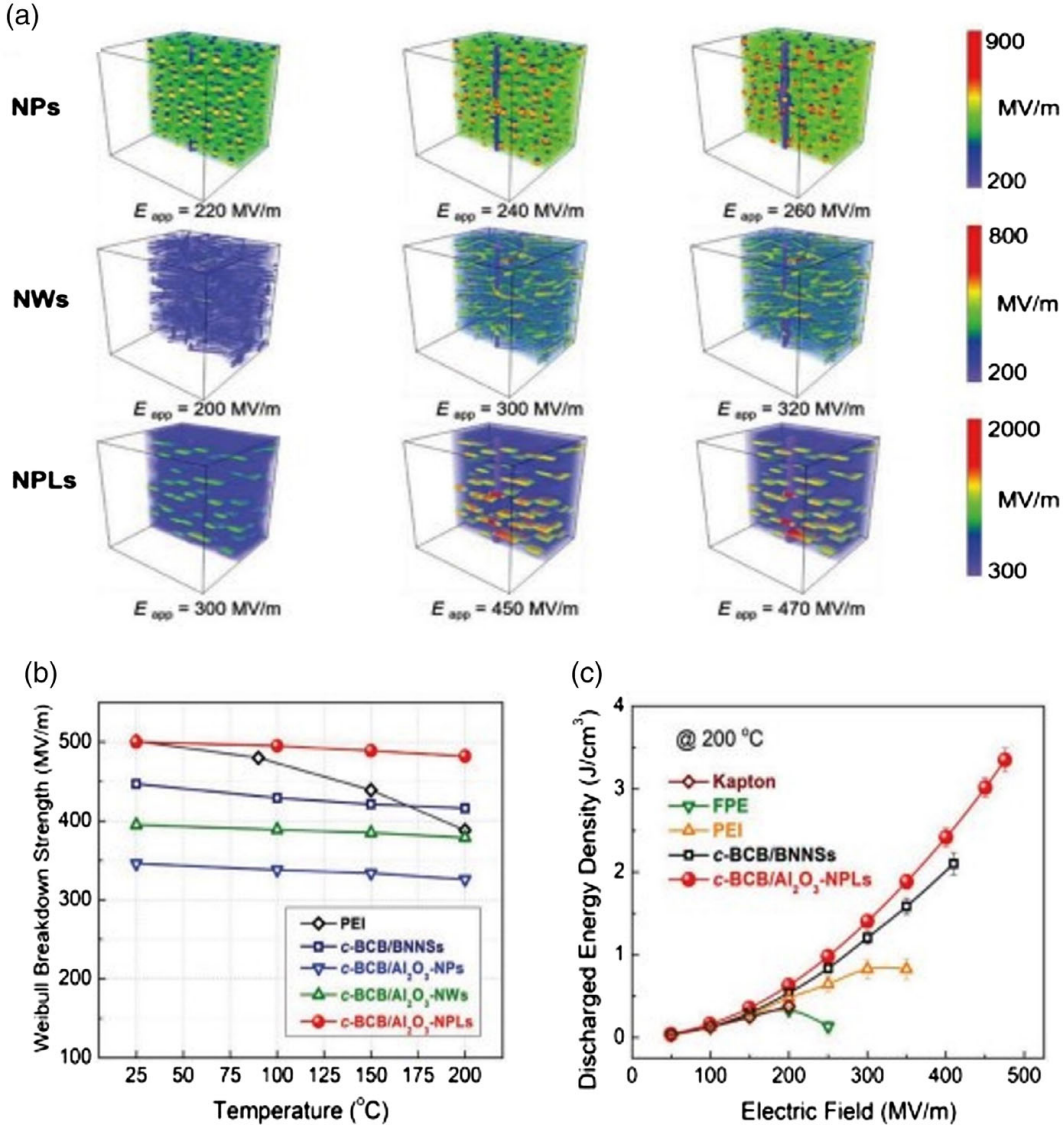
a) The electric field distribution calculated from phase‐field simulations for 7.5 vol% Al_2_O_3_ NPs, NWs, and NPLs in c‐BCB nanocomposites at 150 °C with varying applied electric fields. b) Temperature‐dependent Weibull breakdown strength at 150 °C for pure PEI and nanocomposites. c) Charge–discharge efficiency and discharged energy density of high‐temperature dielectric polymers and c‐BCB nanocomposites at 200 °C. Reproduced with permission.^[^ ^29^ ^]^ Copyright 2019, Wiley‐VCH.

Further, they observed that the expected maximum breakdown strengths occur at roughly 310, 390, and 480 MV m^−1^ for composites containing NPs, NWs, and NPLs, respectively (**Figure** **10**a), and are consistent with the experimental findings. The applied electric field is found to be most uniformly distributed throughout the polymer matrix by NPLs. The electric fields are concentrated around the NPs and the vertices of the NWs, increasing breakdown routes and compromises *E*_b_, leading to an increase in breakdown rates. As shown in Figure 10b, the Al_2_O_3_‐based polymer composites exhibited remarkable stability in their breakdown strength (*E*_b_) as temperature varies. For instance, the breakdown strength of c‐BCB/ Al_2_O_3_‐7.5 vol% NPLs only reduces by 3.6%, from 500 to 482 MV m^−1^, as opposed to a 6.9% decline for c‐BCB/BNNSs, from 447 to 416 MV m^−1^, when the temperature increased from 25 to 200 °C. The breakdown strength of polyetherimide (PEI), a top high‐temperature dielectric polymer, decreases noticeably with temperature, from 501 MV m^−1^ at 25 °C to less than 400 MV m^−1^ at 200 °C. The breakdown strength is improved by the crosslinking of BCB with the polymer matrix and good compatibility between the fillers and polymer matrix reduces the possibility of interfacial defects. However, any further increase of Al_2_O_3_ fillers to c‐BCB reduces the breakdown strength, most likely as a result of filler aggregation.^[^ ^29^ ^]^ Al_2_O_3_‐based polymer composites perform exceptionally well in terms of breakdown strength stability at high temperatures (*E*_b_). When the temperature was raised from 25 to 200 °C, the *E*_b_ of c‐BCB/Al_2_O_3_‐7.5 vol% NPLs decreased by just 3.6%; however, polyetherimide showed a considerable decline in the same temperature range, as shown in Figure 10c. This demonstrates that Al_2_O_3_ filler addition enhances interface compatibility and enhances *E*_b_. However, the loss increases at higher temperatures due to diverse conduction pathways. c‐BCB/Al_2_O_3_‐NPL outperforms other polymer composites and high‐temperature dielectric polymers in terms of energy density and charge–discharge efficiency at high electric fields and high temperatures.^[^ ^29^ ^]^

#### 4.3.4 Ca_2_Nb_3_O_10_ Nanosheets (CNOs)

Ca_2_Nb_3_O_10_ has a complex crystal structure and Ca_2_Nb_3_O_10_ nanosheets (CNOs) are typically synthesized by delaminating precursor‐layered perovskite KCa_2_Nb_3_O_10_.^[^ ^87^ ^]^ Bao et al. outlined a method for adding negatively charged CNOs to polymer‐based dielectrics to enhance their breakdown strengths and energy storage capabilities.^[^ ^88^ ^]^ In both poly(vinylidene fluoride) and polystyrene‐based nanocomposites, the breakdown strength (792 MV m^−1^) and the energy density (36.2 J cm^−3^) have significantly increased, surpassing previous records for flexible polymer‐based dielectrics. This was explained through phase‐field simulations that demonstrated how negatively charged nanosheets and positively charged polyethyleneimine interact together to create a local electric field that suppresses secondary impact‐ionized electrons and obstructs the breakdown paths in the nanocomposites.

**Figure 11:**
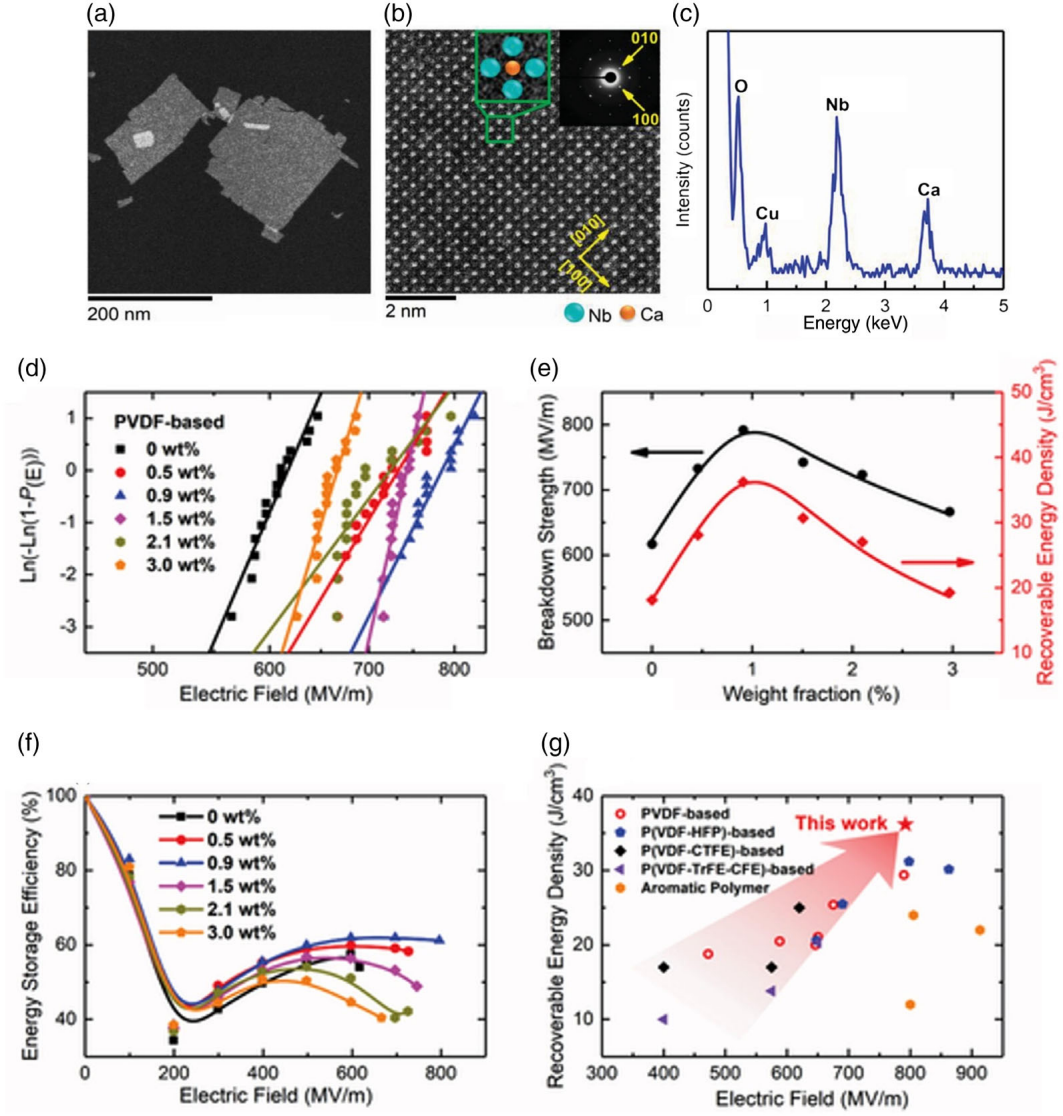
a) TEM image of CNO nanosheets on an ultrathin copper mesh. b) HAADF image of a CNO nanosheet. The inset shows the electron diffraction pattern of the selected area. c) EDS pattern of CNO nanosheets on an ultrathin copper mesh. d) Weibull distribution of breakdown strengths of nanocomposites made of PVDF. The fitting outcomes using a two‐parameter Weibull distribution function are represented by the solid lines. e) Variation of breakdown strength (left) and the maximum recovered energy density (right) as a function of w% of CNO nanosheets, and f) Energy storage effectiveness as a function of applied electric field for PVDF‐based nanocomposites. g) Maximum recoverable energy densities of nanocomposites made of PVDF, P(VDF‐HFP), P(VDF‐CTFE), P(VDF‐TrFE‐CFE), and aromatic polymers with respect to the applied field. Reproduced with permission.^[^ ^88^ ^]^ Copyright 2020, Wiley‐VCH.

The TEM picture shows that CNO nanosheets can have lateral sizes of up to several hundred nanometers (**Figure** **11**a). The atomic structure of CNO is clearly visible in the high‐angle annular dark‐field (HAADF) picture, and the locations of the Nb and Ca atoms are indicated in the inset. The electron diffraction pattern and the EDS analysis for the selected region show that the CNO nanosheet is highly crystalline (Figure 11b). The distinct (001) peaks in the X‐ray diffraction (XRD) patterns suggested the layered structure of CNO nanosheets, providing further evidence that the sheets form layers after drying (Figure 11c).

Unmodified CNO nanosheets are negatively charged, as shown by a surface potential decrease of around 40 mV in CNO nanosheet‐containing regions, as detected by scanning Kelvin probe microscopy (SKPM) (Figure 11d,e). Each surface has a surface charge density of −0.053 C m^−2^ as measured by the titration approach. It is challenging for the CNO nanosheets to detach from the colloid due to their negative change as it causes electrostatic repulsion between them. To resolve this, the colloid was mixed with the cationic surfactant polyethyleneimine (PEI), which produced electrically neutral and precipitable PEI/CNO/PEI nanosheets due to electrostatic interactions. According to the Helmholtz electric double‐layer model, each PEI layer has a positive charge density of +0.053 C m^−2^. The protonated amine groups in PEI, which are readily able to form bonds with H^+^ ions in an aqueous solution, are the sources of this positive charge. The presence of nitrogen in the energy‐dispersive spectroscopy (EDS) analysis (Figure 11f) and the appearance of the feature band at 1627–1697 cm^−1^ in the infrared spectra (Figure 11g), which represents positively charged NH^3+^ groups and primary amine groups in PEI, can both be used to identify the presence of PEI in the CNO nanosheets sandwiched with PEI. The strong band at 550–730 cm^−1^ suggests that the distortion of NbO_6_ octahedra has decreased or that there are more bridging oxygens present. Atomic force microscopy (AFM) was used to determine the thickness of the PEI/CNO/PEI nanosheets, which were approximately 3.4 nm, with a monolayer CNO nanosheet having a thickness of 1.5 nm and two PEI layers having a thickness of 0.95 nm each.

#### 4.3.5 Titanium Dioxide (TiO_2_)

The performance of energy storage in dielectric polymer nanocomposites is improved by the introduction of 2D high‐*k* titanium dioxide nanosheets. Zhu et al. fabricated TiO_2_ polymer nanocomposites exhibiting high‐*k* and lower dielectric loss compared to that of the pure polymer.^[^ ^5^ ^]^ They reported an energy density of 13.0 J cm^−3^ for the nanocomposite containing 5% 2D nanosheets, which is four times higher than the energy density of widely used BOPP polymer. Additionally, the study contrasts nanocomposites with 0D and 1D nanofillers and discovers that the 2D nanofiller is superior in advancing energy storage in polymer nanocomposites. The electric field distribution in the nanocomposites with different nanofillers was also studied through finite element simulation.^[^ ^5^, ^89^, ^90^ ^]^

### 4.4 Hybrid 2D‐Nanofiller‐Based Polymeric Dielectric Capacitors

**Figure 12:**
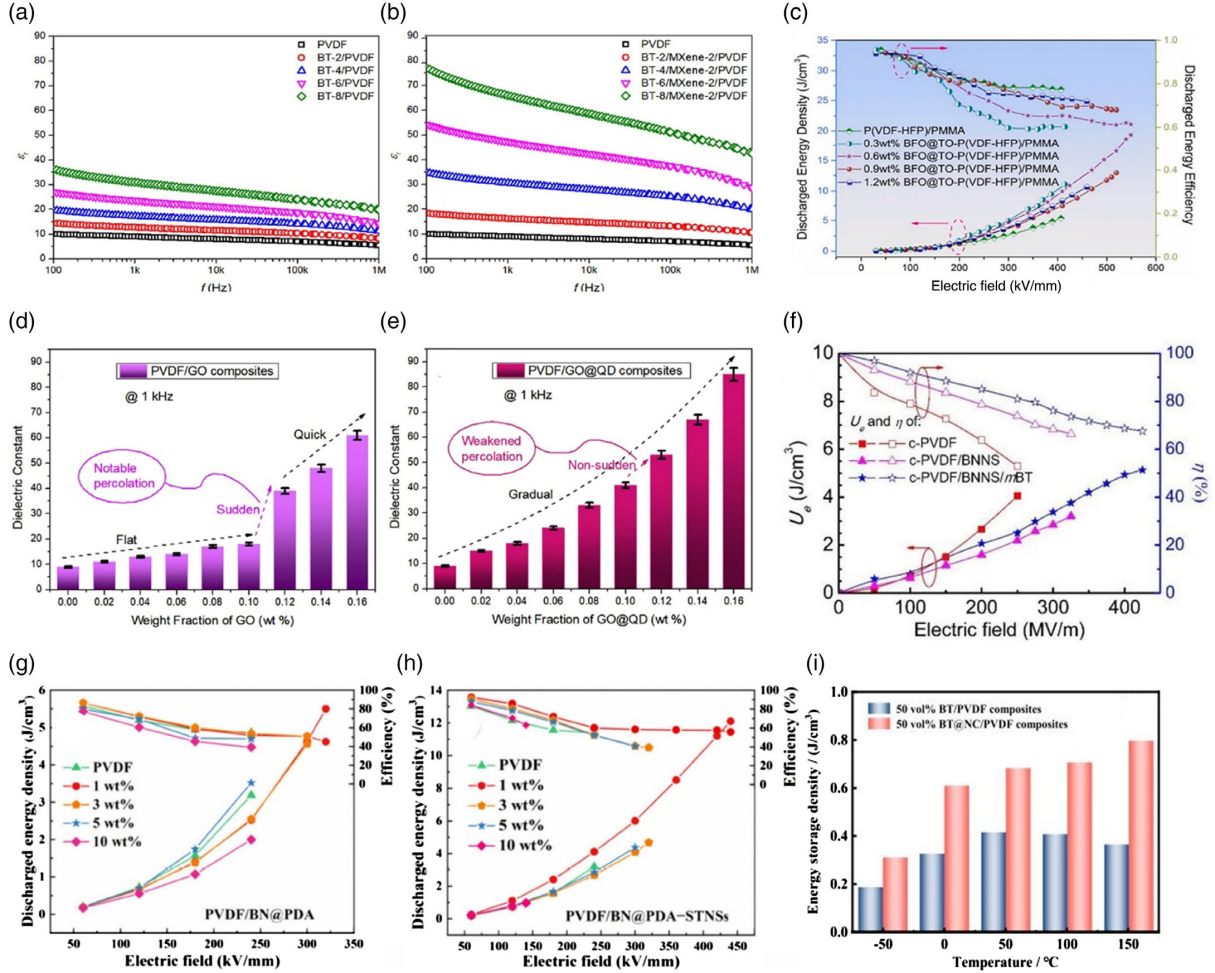
a,b) Variation of the dielectric constant with the frequency of binary (a) and ternary (b) composites. Reproduced with permission.^[^ ^25^ ^]^ Copyright 2019, Elsevier. c) Variation of the discharged energy density and efficiency of BFO@TO‐P(VDF‐HFP)/PMMA composites. Reproduced with permission.^[^ ^93^ ^]^ Copyright 2022, MDPI. d,e) Variation of the dielectric constant of binary and ternary composites with wt% of GO. Reproduced with permission.^[^ ^91^ ^]^ Copyright 2020, Elsevier. f) Variation of the discharged energy density and efficiency of binary and ternary composites as a function of field. Reproduced with permission.^[^ ^97^ ^]^ Copyright 2018, Elsevier. g,h) Variation of discharged energy density and efficiency of PVDF/BN@PDA and PVDF/BN@PDA‐STNS. Reproduced with permission.^[^ ^93^ ^]^ Copyright 2022, MDPI. i) Energy storage density of BT/PVDF composites and BT@NC/PVDF with 50 vol% fillers. Reproduced with permission.^[^ ^95^ ^]^ Copyright 2022, Elsevier.

The incorporation of more than one 2D nanofiller into polymer nanocomposites can enhance the energy storage capacity in various ways as reported in recent articles.^[^ ^25^, ^91^, ^92^, ^93^ ^]^ For example, Feng et al. followed a solution cast process to prepare ternary BT/MXene/polymer nanocomposites with 8 wt% of BT and 2 wt% of MXene that exhibited a huge enhancement of dielectric constants up to 77 with a low dielectric loss of 0.15 at 100 Hz, as shown in **Figure** **12**a,b.^[^ ^25^ ^]^ In another study, they fabricated binary (PVDF/GO) and ternary (PVDF/GO/ nitrogen‐doped Ti_3_C_2_ MXene quantum dots polymer/hybrid nanocomposite films and observed that ternary nanocomposite films had improved dielectric constants and breakdown strengths compared to binary nanocomposites.^[^ ^91^ ^]^ Figure 12d,e displays the variation of the dielectric constant as a function of w% of GO for the polymer‐binary and GO + MXene quantum dots for the ternary nanocomposites.

Doping synergistic dual‐fillers titania nanosheets (STNSs) and boron nitride sheets into polydopamine and poly(vinylidene fluoride) (PVDF) polymers showed remarkable enhancement in dielectric properties, as reported by Zhu et al.^[^ ^93^ ^]^ They observed a high dielectric constant of 13.9 at 1 Hz and a *U*_e_ of 12.1 J cm^−3^ at 440 kV mm^−1^, for filler concentrations of 1 wt% BN@PDA and 0.5 wt% STNSs (1 wt% PVDF/BN@PDA‐STNSs), as shown in Figure 12g,h. The simulation results show that utilizing BN@PDA sheets increases breakdown strength while incorporating STNSs enhances polarization, matching experimental results. This offers a systematic approach to design energy storage dielectrics with improved dielectric properties.^[^ ^93^ ^]^

Jing et al. fabricated a hybrid nanofiller/polymer nanocomposite by adding a small amount of BiFeO_3_@TiO_2_ (BFO@TO) into P(VDF‐HFP)/PMMA blended polymer, leading to an outstanding energy density of 19.3 J cm^−3^ at 549.2 kV mm^−1^ as shown in Figure 12c.^[^ ^92^ ^]^ Zhang et al. reported a trilayer architecture consisting of nanofillers of BaTiO_3_ (BT) and CNT with polyimide (PI). The (BT)/PI‐CNT/PI‐ BaTiO_3_/PI film showed an increase of dielectric constants by 700% compared to pure PI at 10 kHz without compromising the low loss tangent values of PI, for the composite with 50 wt% of BT and 4 wt% of CNT.^[^ ^94^ ^]^

Zhu et al. incorporated BaTiO_3_ modified with Nb_2_O_5_ and Co_3_O_4_ (BT@NC) to PVDF and prepared BT/PVDF and BT@NC/PVDF nanocomposites.^[^ ^95^ ^]^ They observed a high dielectric constant of 70.2 (at 104 Hz) with a low dielectric loss of 0.02 for the composite with 50 vol% of BT@NC. This modified filler has also improved the energy density by more than 123.2% than that of 50 vol% BT/PVDF composites.^[^ ^96^ ^]^

Peng et al. developed Mo@MoO_3_/PVDF composites that showed high dielectric constants and low dielectric losses. They fabricated the composite by encapsulating molybdenum (Mo) particles with a thin layer of MoO_3_ and incorporating the resulting Mo@MoO_3_ particles with PVDF polymer.^[^ ^96^ ^]^ Xie et al. reported a huge improvement in energy density by introducing BNNS into PVDF‐based BaTiO_3_ (mBT) binary composites. The ternary composite with 6 wt% of BNNS and 5 wt% of mBT presented a high breakdown field (*E*_b_) of 400 MV m^−1^ and an energy density of 5.2 J cm^−3^, which is superior to their binary composites.^[^ ^97^ ^]^

Chen et al. prepared BT@MgO/P(VDF‐HFP) nanocomposites by employing MgO as a buffer barrier to mitigate the mismatched dielectric characteristics between BT nanoparticles and poly(vinylidene fluoride‐hexafluoropropylene) (P(VDF[1]HFP)). The BT@MgO/P(VDF‐HFP) nanocomposite with a 1% vol filling ratio exhibited a maximum energy density of 5.6 J cm^−3^ which is higher than that of the host polymer and BT‐filled counterpart with the same filler amounts.^[^ ^98^, ^99^ ^]^

## 5 Polymer 2D Nanocomposites for High‐Temperature Dielectric Capacitors

Dielectric capacitors are well‐established components of energy storage devices due to their exciting features like easy transportability, lightweight, mechanical flexibility, scalability, low cost, and environment‐friendly nature. However, instability and low energy density (*U*_e_) of the capacitors at high temperatures hinder their application at the industrial level. The commercially used biaxially oriented polypropylene (BOPP) operates at 120 °C giving *U*_e_ of 0.27 J cm^−3^ with a charge–discharge efficiency of 90%.^[^ ^100^ ^]^ Currently, electric vehicles, aircraft, turbine engines, and electronic sensing devices need capacitor‐based energy devices that can operate above 200 °C.^[^ ^101^, ^102^, ^103^, ^104^, ^105^ ^]^ As the temperature increases above the glass transition temperature (*T*_g_), polymers start to lose their stability. Moreover, the charge–discharge efficiency of polymer dielectric films at higher electric fields and elevated temperatures decreases due to the increase of leakage current, leading to the reduction of the amount of charge stored in the capacitor and hence the efficiency of the device. To be employed in extreme settings, dielectric capacitors must be able to withstand both high temperatures and strong electric fields to maintain their higher efficiency. Researchers adopted an efficient path to address this issue by introducing high bandgap nanofillers such as BNNS, MXene, Al_2_O_3_, HfO_2_, and TiO_2_ into polymers such as polyimide (PI).^[^ ^60^, ^106^, ^107^, ^108^, ^109^ ^]^

**Figure 13:**
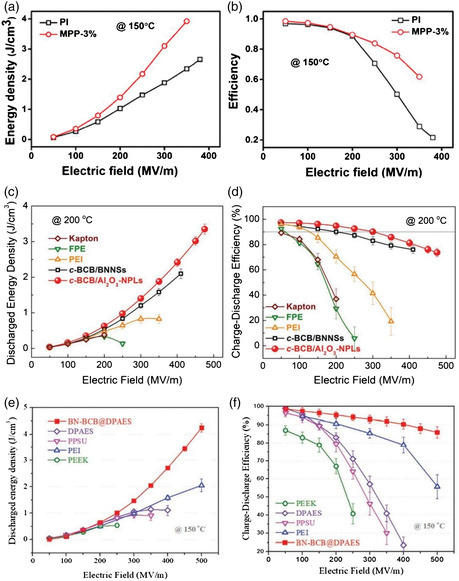
a,b) Energy density and charge–discharging efficiency of pure polyimide (PI) and MoS_2_‐g‐PMMA/PI (MPP‐3%) films at 150 °C. Reproduced with permission.^[^ ^109^ ^]^ Copyright 2021, Wiley‐VCH. c,d) Discharged energy density and charge–discharge efficiency of cyclic divinyltetramethyldisiloxane‐bis(benzocyclobutene) (BCB), fluorene polyester (FPE), polyetherimide (PEI), cBCB/BNNS and c‐BCB/Al_2_O_3_ nanoplates as a function of electric fields at 150 °C. Reproduced with permission.^[^ ^29^ ^]^ Copyright 2019, Wiley‐VCH. e,f) Discharged energy density and charge–discharge efficiency for c‐BC/BNNS, FPE, and Kapton at 200 and 250 °C. Reproduced with permission.^[^ ^27^ ^]^ Copyright 2015, Springer Nature.

Li et al. fabricated a polymer nanocomposite by coating 2D MoS_2_ nanosheets with a thin layer of PMMA and then mixing with polyimide (PI) solution.^[^ ^109^ ^]^ Although the dielectric constant of the PMMA/PI (MPP‐3%) was not improved significantly, the high breakdown strength enables a higher energy density of 8.6 J cm^−3^ at room temperature and 3.92 J cm^−3^ at 150 °C, as shown in **Figure** **13**a. At the same elevated temperature, the nanocomposite film showed an efficiency (*η*) of 61.7%, as shown in Figure 13b. The study conducted by Li et al. observed that at 200 °C, c‐BCB/Al_2_O_3_ nanoplates polymer composite with the 7.5 vol% of Al_2_O_3_ exhibits a higher energy density of *U*_e_ of 3.02 J cm^−3^ with a *η* of 76.1% at 450 MV m^−1^, as shown in Figure 13c,d.^[^ ^29^ ^]^ Xu et al. anchored poly (aryl ether sulfone) (DPAES) on the surface of functionalized BN‐BCB to prepare BN‐BCB@DPAES nanocomposites which result in the improvement of breakdown strength (*E*_b_), discharged energy density (*U*_e_), and charge–discharge efficiency (*η*) at elevated temperatures. They recorded a discharged energy density of 4.2 J cm^−3^ for 500 MV m^−1^ at 150 °C as depicted in Figure 13e,f.^[^ ^27^ ^]^

In another study, Li et al. fabricated c‐BCB/BNNS nanocomposites that displayed improved high‐voltage capacitive energy storage capabilities with a Weibull breakdown strength of 403 MV m^−1^ and an energy density of 1.8 J m^−3^ at 250 °C.^[^ ^72^ ^]^ They further enhanced the energy density of the polymer up to 4 J cm^−3^ at 150 °C by making a trilayered polymer nanocomposite by sequential casting c‐BCB/BNNS, c‐BCB/BT, and c‐BCB/BNNS with a thickness ratio of 1:2:1, respectively.^[^ ^99^ ^]^

## 6 Mechanical Stability of Polymer 2D Nanocomposites for Film Dielectric Capacitors

The use of 2D nanosheets is expanding rapidly along with the ongoing development of the production process. Compared to 0D and 1D nanofillers, 2D nanosheets possess a lower dielectric loss and greater breakdown strength.^[^ ^110^, ^111^, ^112^ ^]^ Typically, 2D nanosheets are utilized due to their capability to increase the breakdown strength (*E*_b_). Hexagonal boron nitride (h‐BN), an insulator with a broad bandgap (*E*_g_) and an *E*_b_ of 800 MV m^−1^, has a layered structure similar to graphite. As a result, hexagonal boron nitride nanosheets (BNNS) have the potential to significantly enhance the *E*_b_ of the composite. Further, its outstanding mechanical and insulating qualities have the potential to minimize the loss. Wang et al. created a film using BNNS and regenerated chitin (RCH).^[^ ^113^ ^]^ They reported a high breakdown strength of 451 MV m^−1^ for the composite film with 6 wt% of BNNS loading and observed a drop in the dielectric loss from 0.021 to 0.018 at 1000 Hz. In addition, Li et al.^[^ ^27^ ^]^ created P(VDF‐TrFE‐CFE)/BNNS nanocomposites using ferroelectric P(VDF‐TrFE‐CFE) terpolymers combined with very thin BNNS.^[^ ^27^, ^106^, ^114^, ^115^, ^116^ ^]^ In addition, the nanocomposites have been fabricated by attaching poly (aryl ether sulfone) (DPAES) to the functionalized BNNS's (BN‐BCB) surface that leads to a higher charge–discharge efficiency that is greater than 90% at a temperature of 150 °C, with an enhanced energy density of 2.7 J cm^−3^ at 400 MV m^−1^.^[^ ^29^, ^117^ ^]^

Multiphase 2D nanofillers can be incorporated into the polymer matrix as single‐layered or multilayered composites to further increase the energy density. The 2D multiphase systems having conductive 2D fillers and insulating fillers allow the achievement of both higher breakdown and lower loss tangents. By incorporating hybrids of carbon nanostructures and graphene nanoplatelets, thermoplastic polyurethane composites can be fabricated which exhibited enhanced dielectric characteristics.^[^ ^118^ ^]^ Further, the addition of ultra‐small silver nanoparticles successfully reduced the conductivity and dielectric loss of TPU/GNS/Ag composites, which enhanced the composite's ability to store energy.^[^ ^119^ ^]^ In multiphase systems, BNNS and MMT are the dominant insulating 2D fillers to fabricate PEI‐based nanocomposites. These nanocomposites have improved breakdown strengths and dielectric constants that translate to exceptional electrical storage and discharging performance compared to pure PEI. Additionally, compared to nanocomposites containing either nanomontmorillonite (Na+ MMT) or ionic liquid (IL), the PVDF/IL/MMT nanocomposite shows better energy storage ability. By the rational alternation of the insulating BNNS layers with the highly aligned conductive reduced graphene oxide (rGO), Guo et al.^[^ ^120^ ^]^ created a unique micro sandwich structured nanocomposite. The electrical conductivities of rGO and BNNS differ, and by alternately stacking them, they may provide a higher dielectric constant with a low breakdown strength. With a high dielectric constant, a higher energy density, and an outstanding thermal conductivity at a modest hybrid filler percentage of 2.5 vol%, the nanocomposite outperforms pure polymer films.^[^ ^120^ ^]^

**Figure 14:**
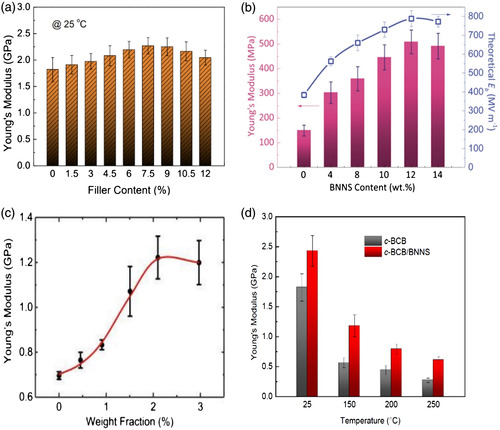
a) Variation of Young's modulus of c‐BCB/Al_2_O_3_ nanocomposites with the change in filler content percentage at room temperature. Reproduced with permission.^[^ ^29^ ^]^ Copyright 2019, Wiley‐VCH. b) Variation of Young's modulus and the theoretical predictions of breakdown strength of P(VDF‐TrFE‐CFE)/BNNS nanocomposites as a function of filler percentage. Reproduced with permission.^[^ ^27^ ^]^ Copyright 2015, Springer Nature. c) Variation of Young's modulus of CNO‐based nanocomposite with change in percentage of CNO nanosheet in the nanocomposite. Reproduced with permission.^[^ ^88^ ^]^ Copyright 2020, Wiley‐VCH. d) Young's modulus of c‐BCB and c‐BCB/BNNS at different temperatures with 10% BNNS content. Reproduced with permission.^[^ ^27^ ^]^ Copyright 2015, Springer Nature.

Due to their low operating temperatures, polymer dielectrics cannot withstand extreme conditions that exist in electric vehicles and underground exploration of natural gas and oil. However, Li et al. reported stable dielectric characteristics across a wide range of frequencies and temperatures for crosslinked polymer nanocomposites containing BNNS.^[^ ^27^ ^]^ Their nanocomposites displayed exceptional high‐energy storage capacities at high temperatures. Further, their thermal conductivity has significantly improved due to the inclusion of BNNS, which promotes heat dissipation compared to pure polymers. These nanocomposites had outstanding dielectric and capacitive performance even after repeated bending cycles. Also, they were lightweight, photo‐patternable, and structurally flexible. To create cross‐linked c‐BCB/BNNS nanocomposites, bis(benzocyclobutene) (BCB) was thermally crosslinked in the presence of BNNS. Further, they investigated the relationship of the dielectric constant (*k*), and the dissipation factor (DF) to the temperature and the frequency.^[^ ^27^ ^]^ While FPE, the study's second‐best dielectric, exhibited a change in dielectric constant (*k*) by almost 8% at 300 °C compared to that at the room temperature, c‐BCB/BNNS exhibited a minimal fluctuation for the same temperature range at 104 Hz, which is the frequency of conventional power conditioning. For the temperature range of 25–300 °C, the dielectric constant's temperature coefficient for c–BCB/BNNS was approximately 65 ppm per °C, compared to 308 ppm per °C for FPE and 498 ppm per °C for Kapton. The variation of the dielectric constant of c‐BCB/BNNS is just 1.6% at 250 °C even under a direct current bias voltage of 50 MV m^−2^, as opposed to 8.5% for FPE. Upon increasing the temperature to 300 °C, the dissipation factor (DF) of c‐BCB/BNNS increased from 0.09% to 0.13% at 104 Hz. Although Kapton exhibited similar stability of direct current bias with temperature, a significant increment in direct‐current bias was seen in other polymer dielectrics. Evidently, c‐BCB/BNNS gives the most stable dielectric constant and DF at the frequencies of 102–106 Hz at higher temperatures out of all the dielectrics evaluated. At room temperature, a comparison of Young's modulus was done by changing the percentage of fillers in the c‐BCB/Al_2_O_3_ nanocomposite, and the highest value was observed when the filler content was 7.5%, as shown in **Figure** **14**a.

Similar to Bao et al.'s^[^ ^88^ ^]^ work, Li et al.^[^ ^27^ ^]^ designed a set of experiments to study the dielectric and mechanical properties of P(VDF‐TrFE‐CFE)/BNNS, a ferroelectric terpolymer/BNNS composite. In addition to its dielectric properties, its mechanical strength was assessed by studying the variation of Young's modulus with the filler content. As manifested in Figure 14b, Young's modulus increased as the BNNS content was increased reaching a maximum with 12% of filler content, but slowly decreased as the filler percentage was increased. In addition, theoretical values of breakdown strength have been plotted against the filler percentage of the nanocomposite on the same plot.

According to studies by Bao et al.,^[^ ^88^ ^]^ adding negatively charged Ca_2_Nb_3_O_10_ (CNO) nanosheets would increase their breakdown strength and energy density. Poly(vinylidene fluoride)‐based nanocomposite capacitors have the highest energy density of 36.2 J cm^−3^ and a significantly improved breakdown strength of 792 MV m^−1^. These improvements in breakdown strength and energy density serve as proof of concept that can be generalizable. Phase‐field simulations show that the local electric field created by the negatively charged CNO nanosheets sandwiching the positively charged polyethyleneimine is responsible for the further enhancement of the breakdown strength. Bao et al.^[^ ^88^ ^]^ assessed Young's modulus of the CNO‐based nanocomposites and compared them to pure PVDF. As shown in Figure 14c, Young's modulus increased with the filler content, reaching a maximum at 2.1 wt% of CNO nanosheets.

**Figure 15:**
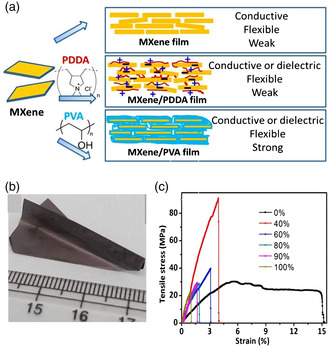
a) Schematic depiction of the work done by Ling et al. that describes the properties of pure MXene films and their polymer composites. b) A demonstration of the flexibility of the synthesized MXene–PVA composite film by folding it into a structure of an airplane. c) Variation of the tensile stress as a function of strain for different MXene–PVA composites, which were synthesized by varying the weight ratio of MXene to PVA. Reproduced with permission.^[^ ^57^ ^]^ Copyright 2014, The Authors, published by National Academy of Sciences, USA.

Similarly, to explore MXene's potential, Ling et al. mixed Ti_3_C_2_T_*x*_ with either PVA which is electronically neutral, or charged PDDA (**Figure** **15**a), which forms the MXene–polymer composite. Ling et al. concluded that the synthesized composites were flexible and had high electrical conductivities with high tensile strengths compared to that of pure PVA.^[^ ^57^ ^]^ In addition to the mechanical strength enhancement, the composite's flexibility increased due to the polymer's intercalation between the MXene flakes. As shown in Figure 15b, cationic intercalation also increased due to the volumetric capacitance of the composite which was at appreciable levels. A Ti_3_C_2_T_*x*_ film that was approximately 3.3 μm thick had tensile strengths of 22 ± 2 MPa and Young's modulus of 3.5 ± 0.01 GPa. An increment of 34% in tensile strength was found when 10% PVA was added. A high tensile strength of 91 ± 10 MPa has been achieved for Ti_3_C_2_T_*x*_/PVA films with 60% weight of PVP had an enhanced, a fourfold increment to the pure Ti_3_C_2_T_*x*_ film (Figure 15c). A 13 μm thick PVA film was found to have a tensile strength of 30 ± 5 MPa. The overall improvement in these films’ stiffness and strength suggests that stress is transferred to the embedded Ti_3_C_2_T_*x*_ nanosheets. This also indicates that there was an interfacial bonding between PVA and nanosheets. Here, OH groups’ termination of the Ti_3_C_2_T_*x*_ had a significant effect in improving these mechanical properties. Furthermore, by adjusting the Ti_3_C_2_T_*x*_‐to‐PVA ratio, Young's modulus of Ti_3_C_2_T_*x*_/PVA films may be customized. In addition, mechanically strong hollow cylinders were fabricated by rolling Ti_3_C_2_T_*x*_ sheets with a thickness of 4–5 μm and by joining the overlapping edges with PVA. A hollow Ti_3_C_2_T_*x*_ cylinder of 6 mm in diameter and 10 mm in height can hold around 4000 times its own mass without any noticeable deformation. However, a cylinder with similar dimensions but with 90% weight Ti_3_C_2_T_*x*_/PVA was able to hold approximately 15 000 times its own mass. These findings indicate that Ti_3_C_2_T_*x*_‐based films with outstanding conductivities have appropriate tensile and compressive strengths that can be utilized in structural devices for energy storage.

## 7 Machine Learning and Theory‐Guided Understanding of Polymer–2D‐Nanofiller‐Based Composites for Dielectric Applications

Material science and engineering have become progressively engrossed in employing data‐driven informatics methodologies during the past decade. One sector of advanced materials research that appears to be prepared for informatics‐driven breakthroughs is polymer science and engineering. Materials constructed from polymers can be simple or complicated. Polymers have the potential to demonstrate diverse properties due to atomic‐level interactions, chain packing, etc.^[^ ^121^ ^]^ The tiniest atoms of the periodic table are frequently used to create polymers (the last being an all‐inclusive articulation to seize phase separation, microstructure, crystallinity, and porosity). Polymers are used in both high‐tech and everyday life applications by tailoring the structure to get desired properties. The latest developments in the polymer informatics domain aim to efficiently leverage existing and simulated data with avante‐garde machine learning (ML) algorithms. These methods can be applied to rapidly determine the properties of new polymers and composites. By reversing the characteristic forecasting pathways, these methods allow the relocation of elements that satisfies the specified attributes.^[^ ^121^ ^]^

Several studies depict the process used by ML techniques for the cogent flowchart of polymer dielectrics. To train and test the ML models, a dataset comprising a sufficient number of specimens with notable attributes is generated. The information for this dataset can originate from investigations or elevated calculations.^[^ ^122^ ^]^ A revved‐up high‐performance dataset containing meaningful statistics was drawn from the complete data matrix. The encoding of nanocomposites or polymers with a sequence of computer decipherable symbols, sometimes known as “descriptors” or “fingerprints” is an essential element of ML techniques.^[^ ^123^, ^124^ ^]^ Depending on the targeted characteristics, the chosen properties in fingerprints should be appropriate, i.e., they ought to influence the final attributes, which may include structural and chemical traits or defining factors (such as density and band constitution of states). To represent and delineate the genetic and structural details of polymers, sequential mathematical notations like the simplified molecular‐input line‐entry system (SMILES) are frequently utilized as fingerprints.^[^ ^125^, ^126^ ^]^ By employing the training examples as input, ML models such as kernel‐based regression, decision trees, and neural networks can understand the relationship between desired characteristics and fingerprints, producing a substitute model for forecasting dielectric properties. Additionally, it is possible to explore a wide area of potential materials using inverted representation approaches like ES techniques and propagative models, which significantly speeds up the identification and creation of novel polymers. Moreover, structure‐property linkage analysis approaches including decipherable neural networks, decision‐tree‐based methodologies, and Pearson correlation estimation can be used to pinpoint the essential factors influencing the desired qualities.^[^ ^127^ ^]^

**Figure 16:**
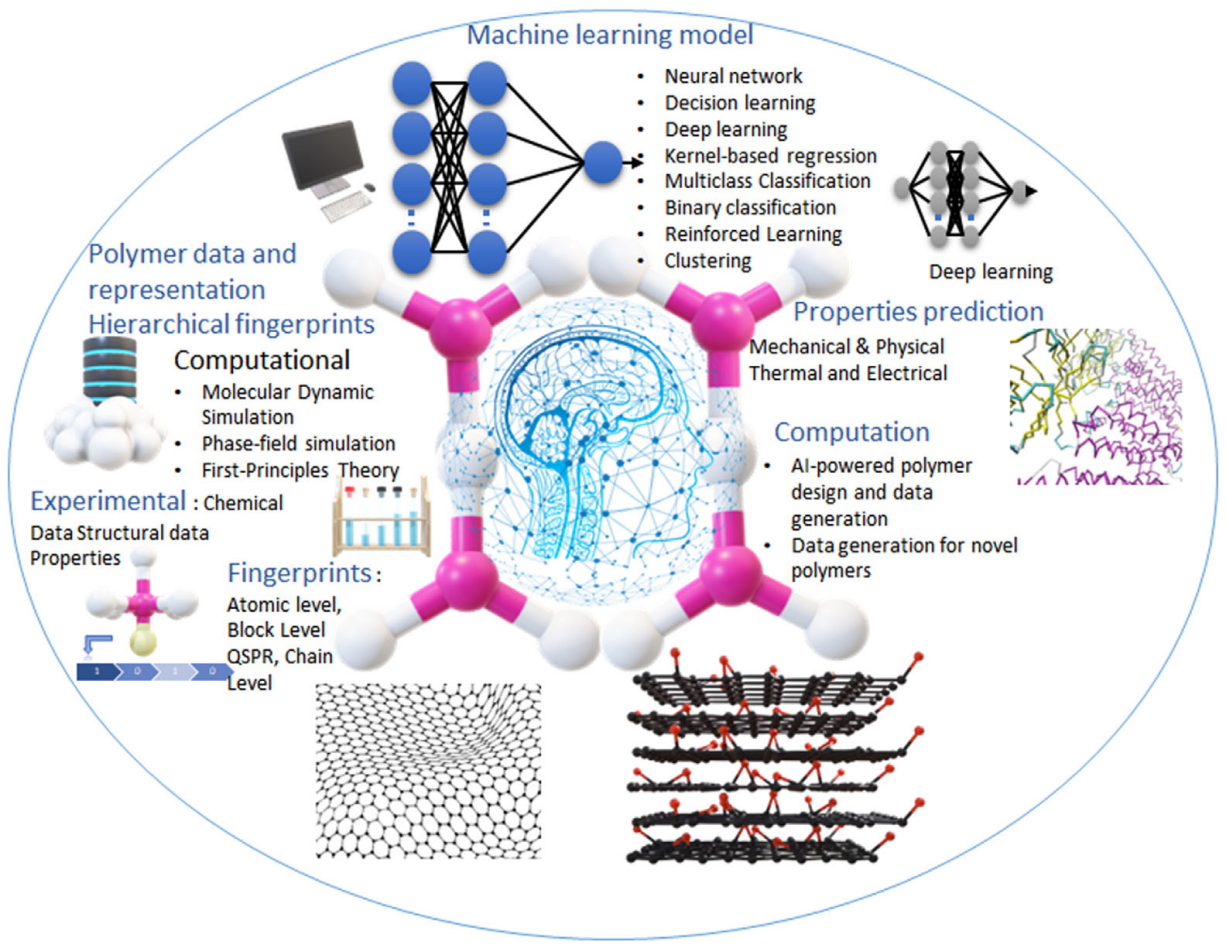
Representation of machine learning techniques utilized to develop novel energy‐storage materials from polymer–nanofiller nanocomposites.

The amount of information on dielectric properties is significantly constrained due to its complex nature. Elevated calculations utilizing rudimentary concepts, numerical simulations, molecular dynamics (MD), stage of evolution model, and finite‐element techniques have been implemented to obtain information, as shown in **Figure** **16**.^[^ ^128^ ^]^ The vast majority of essential dielectric characteristics, such as permeability, loss in conductance, breakdown toughness, glass transmission temperature (*T*_g_), and thermal conductivity, can be either simply determined or indirectly approximated by a set of associated factors. Exhaustive computations (micrometer to millimeter) depending on the phase‐filed model or finite‐element model (FEM) are needed for polymer nanocomposites.^[^ ^129^ ^]^ To examine the disintegration performance and efficient permeability of polymer nanomaterials, phase‐field models are created. In these models, the influence of the nanocomposites’ microstructure, including the dimension and positioning of their nanofillers, may be considered. Shen et al. developed an ML technique to assess the energy storage capacity of polymer nanocomposites relying on large‐scale phase‐field calculations of dielectric reactions, charge transfers, and the disintegration mechanism.^[^ ^130^ ^]^ To comprehend the wide‐ranging features, such as space charge transfer and the heat conduction mechanism, numerical and mathematical calculation techniques like FEM are useful. For instance, the space charge dispensation in dielectrics, which is connected with the breakdown robustness, is typically characterized using a bipolar charge transfer framework.^[^ ^131^ ^]^ To evaluate the breakdown toughness of polymer nanocomposites depending on the charge‐capturing impact of the nanofillers, a multiscale modeling technique that integrates ab initio, Monte Carlo, and continuum scales was also suggested.^[^ ^132^ ^]^

Polymer nanocomposites have recently been thoroughly researched to attain the coveted target attributes by incorporating inorganic nanofillers with certain distinct properties.^[^ ^133^ ^]^ The physical characteristics of nanofillers (permittivity, electrical conductivity, bandgap, thermal conductivity, etc.), the characteristics of the nanofiller–matrix interface (trap states, interfacial polarization, shell parameters in the core–shell structures, etc.), and the geometric microstructure of nanofillers (filler shape, volume fraction, distribution, orientation, etc.) all influence the properties of nanocomposites.^[^ ^134^ ^]^ As a result, the majority of currently used fingerprints for nanocomposites are chosen from these variables based on the desired property of interest, a method referred to as physical‐descriptor‐based methodology.^[^ ^135^, ^136^ ^]^ The primary benefit of physical descriptors is that they offer constructive mappings to processing variables and lucid physical apprehension. For example, nanofiller properties such as morphology, permittivity, electrical conductivity, and volume fraction are used to represent nanocomposites.^[^ ^137^, ^138^ ^]^ Then, an ML strategy is designed to assess energy storage capacity. As shown in Figure 16, we created a fingerprint with a thread of characters that consider physical attributes, shape and form, filler distribution, and shell characteristics in structures of core–shell to assess the relationship between the filler doping strategy and the dielectric features.^[^ ^139^ ^]^

Convolutional neural networks (CNNs) have developed rapidly in recent years, and they can directly draw the geometric features of composite materials. This allows for the straight input of 2D cross‐section images of 3D microstructures into CNNs, which can then forecast characteristics such as thermal conductivity.^[^ ^139^, ^140^, ^141^ ^]^ Additionally, some microstructure characterization and reconstruction (MCR) techniques, such as spatial correlation functions (SCFs) and Fourier space representation of SCF (SDF), have attracted a substantial amount of recognition for their ability to depict polymer composites.^[^ ^142^ ^]^ The benefit of MCR is that a hierarchical reconstruction approach can easily be utilized to rebuild the microstructure. Despite the application of some methods to specify polymer nanocomposites, the fundamental mechanisms governing the effects of nanofillers, such as the impact of the nanofiller–matrix interface on dielectric constant and breakdown strength, remain poorly understood.^[^ ^143^, ^144^ ^]^ More enhanced fingerprints are therefore presumed to precisely forecast certain properties. To predict the dielectric reaction, charge transfer, and disintegrating activity of polymer nanocomposites, Shen et al. created a comprehensive phase‐field model. The potential of storage of energy was then assessed using a machine learning approach that was designed using the results of 6615 increased calculations.^[^ ^145^ ^]^ The breakdown toughness of polymer nanocomposites could be greatly increased as a result of the discovery that parallel perovskite nanosheets choose to restrict and then induce charges to relocate together with the interfaces in the *x*–*y* plane. By creating a polymer nanocomposite made of P(VDF‐HFP) and Ca_2_Nb_3_O_10_, these forecasts were also validated. Numerous similar studies open the door to exploiting 2D polymers’ promising potential for a variety of versatile dielectric applications requiring high voltage durability.^[^ ^146^, ^147^ ^]^

**Figure 17:**
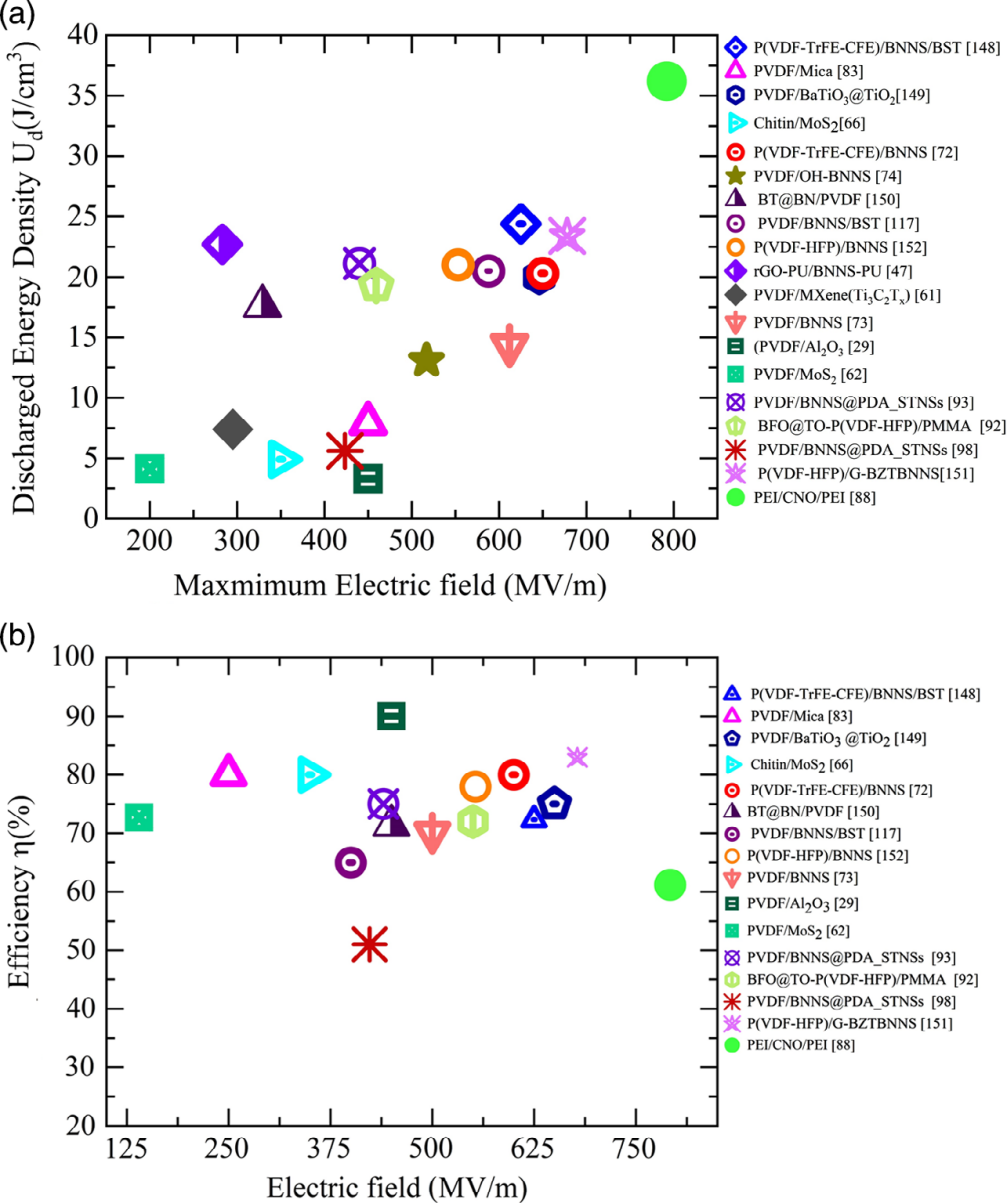
a,b) Comparison of extreme discharge energy densities and efficiencies for polymer–2D‐nanofiller composites at their maximum electric fields.

The energy density and efficiency as a function of the maximum applied electric field of various polymer 2D composite systems are displayed in **Figure** **17**a,b, which were discussed in the previous sections. In addition, we also tabulated the energy density and efficiency of systems including ternary polymer 2D nanocomposites such as P(VDF‐TrFE‐CFE)/BNNS/BST nanocomposites,^[^ ^148^ ^]^ PVDF/BTO@TiO_2_ nanofibers,^[^ ^149^ ^]^ BT@BNNS/PVDF,^[^ ^150^ ^]^ PVDF/G‐BZTBNNS,^[^ ^151^ ^]^ and binary polymer 2D nanocomposite like P(VDF‐HFP)/BNNS (**Table** **1**).^[^ ^152^ ^]^

**Table 1:** Dielectric properties and energy storage capabilities of 2D hybrid nanofiller and polymer composites

Polymer matrix	Filler components	*ϵ*_r_	Tan(δ)	*E* [MV m^−1^]	*U*_d_ [J cm^−1^]^[^ ^3^ ^]^	*η* [%]	Refs.
PVDF	8 wt% BT and 2 wt% MXene	77	0.15	220	7.1	40	[25]
PVDF	3 vol% of BT@TiO_2_	20	0.1	646	20	80	[149]
PVDF	5 wt% BT@BN	12.3	0.08	580	17.66	60	[150]
PVDF	3 vol% BT@SiO_2_	14	0.08	420	11.5	64	[161]
PVDF	4 wt% BT@TiO_2_	13.5	0.32	561.2	21.3	61	[154]
PVDF	6 wt% BNNS and 5 wt% BT	11.8	0.2	400	5.2	83	[97]
PVDF	3.6 vol% BT@ TiO_2_@Al_2_O_3_	12.9	0.05	450	14.84	64.5	[162]
PVDF	2.5 vol% BT@TiO_2_	18	0.1	490	17.6	–	[163]
PVDF	3.47 wt% MoS_2_@MXene	24.3	0.02	400	17.22	66.62	[8]
PVDF	3 vol% BT@ TiO_2_	16.2	0.03	360	10.94	58	[164]
PVDF	2.5 vol% BT/SiO_2_	12.9	0.02	330	6.28	64.3	[165]
PVDF	5 vol% BT@Al_2_O_3_	16.8	0.03	400	12.18	–	[166]
P(VDF–TrFE–CFE)	5 wt% BST and 12 wt% BNNS	42	0.05	625	24.4	72	[148]
P(VDF–HFP	3 vo1% BT @TiO_2_	18	0.08	797.7	31.2	80	[167]
P(VDF–CTFE)	12 wt% BNNS and 15 wt% BT	12.3	0.06	550	22	83	[168]
P(VDF–CTFE–DB)	ZnO@MoS_2_	12.9	0.047	300	7.2	83	[169]
PVDF and PDA	1 wt% BNNS, 0.5 wt% STNS	9.5	0.12	440	12.1	58	[93]
P(VDF–HFP)/PMMA	0.6 wt% BiFeO_3_@TiO_2_	9	0.062	549.2	19.3	61	[92]
PDA/PVDF	1 wt% BT@SiO_2_	12.5	0.01	633	15.4	64	[170]
PEI	3 vol% BNNS‐TiO_2_	3.8	0.02	600	9	90	[171]

## 8 Challenges

One of the limiting factors of dielectric polymers is their low operating temperatures, which is one of the major barriers to their usage in energy storage and conversion in harsh environments commonly present in automobiles, advanced microelectronics, and aerospace power systems. For example, commercially available polypropylene thin film capacitors need to undergo an additional radiator cooling from 140 to 70 °C when used in modern automobiles. Solution‐processed polymer nanocomposite materials composed of polymers and ceramic nanofillers have overcome this high operating temperature barrier, from well below 100 °C to approximately 150 °C,^[^ ^107^, ^153^ ^]^ but further improvement is required for their usage in high‐temperature applications. For example, Li et al. observed an energy density of 1 J cm^−3^ (at 200 MV m^−1^), which remained stable for a broad range of temperatures up to 150 °C for c‐BCB/BNNS polymer nanocomposites.^[^ ^107^ ^]^ The study conducted by Li et al. for BCB/BNNS polymer composites reported an energy density of 1.8 J cm^−3^ at 250 °C at 400 MV m^−1^.^[^ ^27^ ^]^ A recent study by Wang et al. reported a much higher energy density of 3.6 J cm^−3^ for polymer nanocomposites with interposed montmorillonite nanosheets.^[^ ^153^ ^]^ Although these studies conclude that a substantial improvement of both energy densities and working temperatures can be achieved through the integration of 2D materials, the reported energy densities are well below PVDF‐based polymer nanocomposites at room temperature (≈20 J cm^−3^)^[^ ^154^ ^]^ and are on par or below the commercially available BOPP polymer (3.6 J cm^−3^).^[^ ^107^ ^]^

Also, the high dielectric loss due to their elevated dielectric constants (*k*) of 2D material–polymer composites is a major constraint in employing them in practical applications. High dielectric loss is commonly observed in high‐*k* materials due to a variety of reasons from the high level of polarization, slow relaxation, and conducting pathways to strain, and stress. This well‐known paradox is commonly observed in 2D polymer nanocomposites to a higher extent when compared to their polymer counterparts.^[^ ^107^, ^153^, ^155^ ^]^ For example, Tu et al. explored 2D MXene/P(VDF‐TrFE‐CFE) polymer composites and reported a substantial increase in dielectric constant, which however resulted in a significant dielectric loss.^[^ ^26^ ^]^ They reported a nearly sixfold increase in dielectric constant from 55 to 317 from bare polymer to the polymer composite with 4% MXene, but at the same time, the dielectric loss increased by nearly three times from 0.06 to 0.17. Feng et al. reported a large increase in energy density (≈12.5 J cm^−3^) in a PVDF/MXene sandwiched structure despite MXene having a lower breakdown voltage of 150 MV m^−1^ due to its conducting nature.^[^ ^155^ ^]^ However, they observed that the dielectric loss increases as the MXene content increases, which agrees with other studies. Interestingly, when composites made of insulating nanofillers such as mica, TiO_2_, SiO_2_, and Al_2_O_3_, an increase in energy density and dielectric constant have been observed without compromising the charge–discharge efficiency or dielectric loss. Fu et al. reported a threefold increase in energy density from 2.47 J cm^−3^ to nearly 7.93 J cm^−3^ for mica‐PVDF nanocomposites, however, their charge–discharge efficiency was preserved if not slightly increased at higher electric fields.^[^ ^83^ ^]^ The study conducted by Wang et al. also reported a similar observation for mica‐P(VDF‐TrFE‐CTFE)‐g‐PMMA grafted copolymer nanocomposites.^[^ ^84^ ^]^ They observed an 80% increase in energy density from 5.4 to 9.6 J cm^−3^ while preserving the charge–discharge efficiency. 74% efficiency was still retained even at 450 MV m^−1^, the highest field applied to the nanocomposite samples. Li et al. investigated the ferroelectric properties of P(VDF‐HFP) based nanocomposites with SiO_2_, TiO_2_, and Al_2_O_3_.^[^ ^156^ ^]^ They reported observing high energy densities for nanocomposites, with the highest being 15.8 J cm^−3^ for the P(VDF‐HFP)‐Al_2_O_3_ nanoplates while improving charge–discharge efficiencies. Over 80% efficiency has been achieved for the nanocomposite with Al_2_O_3_ nanoplates for fields as high as 700 MV m^−1^. This preservation or improvement of charge–discharge efficiency in nanocomposites is affiliated with the insulating nature of these nanofillers, which function as insulating barriers to resist the current conduction and reduces leakage. This remarkable behavior of nanocomposites of insulating 2D fillers appears to have broken the abovementioned paradox, however, more studies are needed to quantitatively understand these observations.

Another major hurdle in engineering 2D‐polymer nanocomposites is the complexity of interactions at the polymer–nanofiller interface and the lack of understanding of the effects of size, shape, edge effects, and nanofiller volume. For example, composites, where 2D nanofillers are sandwiched between polymers, appear to show better dielectric properties than composites with isotopically dispersed 2D nanoflakes.^[^ ^155^ ^]^ Similarly, composites with 2D nanosheets or nanoplates perform better than those with nanoparticles.^[^ ^156^ ^]^ Although this has been attributed to the in‐plane polarization driven by the shape, further studies are required to quantitatively understand the effect of shapes before employing them in practical applications. Interface science can get further complicated when multiple polymers and multiple 2D materials are involved as theoretical studies are often limited to one polymer and one 2D material.

## 9 Future Research

As mentioned in challenges, understanding complex interactions at the polymer–2D‐flake interface and how they vary from material to material, flake size, thickness, the separation between two flakes, and the orientation is essential for both fundamental science and practical applications. This requires measuring the dielectric properties of individual flakes in the polymer matrix, and observing how their shape, thickness, and orientation impact these properties. This quantitative investigation can be achieved by passing the current through an AFM tip in contact mode (CAFM) through a multi‐step approach. Maruvada et al. illustrated this technique to investigate the breakdown voltage of individual mica nanosheets and their thickness dependence.^[^ ^157^ ^]^ This method can be extended to polymer‐coated 2D flakes by maintaining a thin polymer layer so that individual flakes are visible under the AFM, to study the effects of thickness, size, and orientation on dielectric properties of the hybrid at a level of individual flakes.

In addition to the dimensions of 2D flakes, understanding the role of the thickness of the polymer layer in 2D‐polymer hybrids is essential to investigate interfacial interactions. Thin films have high surface area relative to their volume which may cause improved alignment of polymer chains, increased polarization, and enhanced charge transfer between the polymer and 2D flakes, all of which may improve the overall dielectric performance of the hybrid. Previous studies show that thickness and dielectric properties have a complex relationship, often dictated by the polymer.^[^ ^158^, ^159^, ^160^ ^]^ For example, Neusel et al.^[^ ^158^ ^]^ explored the breakdown voltage for PMMA, PS, PVC, and PE, and concluded a $\frac{1}{\sqrt{d}}$ dependence with the thickness d, for samples above a critical thickness (1 μm) while thin samples displayed no thickness dependence. They attributed this to the existence of conducting surface filaments in thick films that initiate the breakdown. However, the study conducted by Liang et al. shows a decrease in dielectric constant with decreasing film thickness for polyimide thin films, which was explained by the orientation of polymer chains. They found that the leakage current increases when the film thickness is decreased due to the parallel orientation of polyimide chains to the electrodes. These observations suggest that dielectric properties can vary from polymer to polymer with some degree of thickness dependence. So, it is interesting to investigate how this thickness dependence of each polymer impacts the overall performance of polymer nanocomposite thin films.

## 10 Conclusion

2D nanomaterials have a great potential for use in polymer nanocomposites to design high‐energy‐density and high‐power‐density flexible polymeric dielectric capacitors. As shown in studies, 2D nanomaterials can enhance the permittivity and dielectric strength of polymeric nanocomposites, thereby significantly increasing the energy density of nanocomposites. Furthermore, the thermal and mechanical properties of 2D‐nanomaterials‐based nanocomposites can be tuned by tuning the 2D nanomaterial chemistry and polymer–nanomaterial interaction, thereby enabling nanocomposites for harsh conditions. Despite the significant amount of work done on developing and exploring polymer‐2D nanomaterial composites for their potential in high‐energy‐density storage devices, several challenges remain to be addressed such as precise control of the orientation and the thickness of 2D nanosheet within the polymer matrix, 2D nanosheet grafting and the high‐temperature stability to utilize their full potential to revolutionize their use in designing high‐energy‐density dielectric storage devices.

## Conflict of Interest

The authors declare no conflict of interests.
